# Characterisation of the *Gillenia S*-locus provides insight into evolution of the nonself-recognition self-incompatibility system in apple

**DOI:** 10.1038/s41598-025-99335-8

**Published:** 2025-04-26

**Authors:** Ruiling Wang, Cecilia H. Deng, Amandine Cornille, William Marande, Elena López-Girona, Toshi Foster, Deepa Bowatte, Ting-Hsuan Chen, David Chagné, Robert J. Schaffer, Hilary S. Ireland

**Affiliations:** 1 The New Zealand Institute for Plant and Food Research Ltd, Private Bag 92169, Auckland, 1142 New Zealand; 2 Université Paris Saclay, INRAE, CNRS, AgroParisTech, GQE-LeMoulon, 91190 Gif-sur- Yvette, France; 3 Division of Science, New York University Abu Dhabi, Saadiyat Island, Abu Dhabi, United Arab Emirates; 4 INRAE, CNRGV, 31326 Castanet-Tolosan, France; 5 The New Zealand Institute for Plant and Food Research Ltd, Private Bag 11600, Palmerston North, 4442 New Zealand; 6 The New Zealand Institute for Plant and Food Research Ltd, 55 Old Mill Lane, Motueka, 7198 New Zealand; 7 School of Biological Sciences, The University of Auckland, Private bag 91629, Auckland, 1142 New Zealand; 8 The New Zealand Institute for Plant and Food Research Ltd, Private Bag 4704, Christchurch, 8140 New Zealand

**Keywords:** Self-incompatibility, *S*-locus, *Gillenia*, *Malus*, Self-recognition, Nonself-recognition, Plant molecular biology, Self incompatability

## Abstract

Self-incompatibility (SI) in plants has evolved independently multiple times and *S*-RNase-based gametophytic self-incompatibility (GSI) is most common. The Rosaceae family possesses both self-recognition (*Prunus*) and nonself-recognition (*Malus*) GSI systems, and the latter is widespread in flowering plants. *Gillenia trifoliata* is a Rosaceae species related to *Prunus* and *Malus*, providing utility for understanding SI evolution. *Gillenia* is sister taxon to *Malus*, but unlike *Malus*, has not undergone polyploidisation. In addition, the common ancestor of *Gillenia* and *Prunus* is close to the origin of the subfamily. Using a highly contiguous *Gillenia* genome, orthologous regions to both *Malus* and *Prunus S*-loci were identified. Only the *Prunus*-like *S*-locus was highly polymorphic and had signatures of a functional *S*-locus including positive selection of the *S*-RNase. This suggests a self-recognition system controls SI in *Gillenia*, and the common ancestors of *Gillenia* and *Prunus*, and *Gillenia* and the apple tribe, likely had a self-recognition SI system. Comparative genomics between *Gillenia* and *Malus* suggest apple lost the self-recognition mechanism, and a nonself-recognition mechanism evolved independently from a rudimentary locus with at least one male *S*-determinant. Repetitive sequences in the *Malus*-like *S*-locus in *Gillenia* may facilitate illegitimate recombination, suggesting putative mechanisms of evolution of nonself-recognition *S*-loci.

**Supplementary Information:**

The online version contains supplementary material available at 10.1038/s41598-025-99335-8.

## Introduction

Self-incompatibility (SI) is a pre-fertilisation genetic mechanism to promote outcrossing. SI systems have evolved independently at least 35 times in flowering plants^1^ and the gametophytic SI (GSI) system (in which SI is determined by the genotype of the haploid pollen gamete) has the broadest taxonomic range^2–5^. Among GSI systems, the *S*-RNase-based GSI system is the putative ancestral state for core eudicots^6^. In this system, a single highly polymorphic *S*-locus contains a pistil (female) *S*-determinant (*S*-RNase) and pollen (male) *S*-determinant(s) (*S*-F-box) genetically linked within an *S*-haplotype^3^. When active, the pistil *S*-RNase degrades cellular RNA in the pollen tube thus inhibiting growth^7^. The conditions under which the *S*-RNase is active, depends on whether the system utilises a self- or nonself-recognition mechanism (‘self’ referring to the *S*-haplotype). For *S*-RNase-based GSI self-recognition systems, an inactive pistil *S*-RNase becomes cytotoxic upon recognition by a single self-pollen *S*-F-Box, whereas, in the case of nonself-recognition, an active pistil *S*-RNase is detoxified upon recognition by multiple possible nonself-pollen *S*-locus F-Box (SLF) (or *S*-locus F-Box Brothers (SFBB) in Rosaceae)^8,9^. The different genetic architectures of the two mechanisms have major evolutionary consequences: self-recognition systems co-evolve within an *S*-haplotype and rare *S*-haplotypes benefit from negative-frequency dependence (i.e., absence of recognition of rare *S*-alleles increases fitness)^10^; nonself-recognition systems co-evolve within a population, and the effective fitness of negative-frequency dependence also depends on the ‘completeness’ of the multiple nonself SLFs to recognise a wide-range of population *S*-RNases^8,11,12^; mechanisms which contribute to this ‘completeness’ are therefore integral to understanding SI function and evolution^13^.

Phylogenetic analyses of *S*-RNase GSI *S*-genes show that *S*-*RNase* (T2 RNase) and *SLF* genes from species spanning two major clades (superrosids and superasterids) cluster within monophyletic clades suggesting a single origin of each *S*-gene component^4,6,14^. Given nonself-recognition GSI systems are well-characterised in *Petunia* (Solanaceae), *Antirrhinum* (Plantaginaceae), *Citrus* (Rutaceae), and *Malus*, *Pyrus* and *Rosa* (Rosaceae)^9,15–22^, it is often concluded that nonself-recognition with multiple *SLF* factors was the likely ancestral state of the *S*-RNase GSI system in eudicots^4,8,20^. Within Rosaceae, *Prunus* bears a self-recognition *S*-RNase GSI system^17,18^, which is designated on the basis of a distinct *Prunus*-specific *S-locus F-box* (*SFB*) clade within *SLF* phylogenies^23^ and loss-of-function mutations in *SFB* causing self-compatibility^24,25^, among other lines of evidence. It is postulated the *Prunus* self-recognition system evolved independently, and the Rosaceae ancestor had a nonself-recognition system^4,8^, like that of *Rosa* and *Malus*. The *Prunus S*-locus is orthologous to the *S*-loci of *Rosa* and *Fragaria*, and it was recently shown that *Rosa* putatively bears a multiple *SLF* nonself-recognition GSI system while the *Fragaria* recognition mechanism is unknown^20,23,26^. The *Malus* multiple *SFBB* nonself-recognition *S*-locus is paralogous to *Prunus*, and putatively arose through convergent *de novo* evolution^23,27^. Lack of highly contiguous genome assemblies has hampered studies of *S*-locus evolution.

The *Gillenia* genus occupies a phylogenetically informative position within Rosaceae to aid exploration of SI evolution. *Gillenia* is sister taxon to the Maleae tribe comprising approximately 1000 species, among which apple, Japanese pear, and *Sorbus* bear nonself-recognition GSI systems at the same genomic locus^16,21,28^. Studies suggest the Maleae tribe (base chromosome, 17, and rarely 15) evolved from an ancestor similar to *Gillenia* (base chromosome, 9), through autopolyploidisation, aneuploidy, and diploidisation^29,30^, and recently, high synteny was shown between *G. trifoliata* and apple (*Malus domestica*) genomes where 12 (of 17) homoeologous apple chromosomes shared end-to-end co-linearity with six *Gillenia* chromosomes^31^. *Gillenia* therefore provides utility to deconvolute the ancient polyploidisation of the Maleae tribe. Also, the last common ancestor of *Prunus* and *Gillenia* is thought to be close to the origin of the second largest Rosaceae subfamily (Amygdaloideae)^32,33^, providing insight into putative ancestral states. This study aimed to identify and characterise the *S*-locus in *Gillenia* to understand evolution of SI systems, through comparison of *S*-loci between the highly contiguous genomes of *G. trifoliata* and apple root-stock cultivar *Malus domestica* ‘M9’ and informative Rosaceae species.

## Results

### Pollination assays demonstrate self- and cross-incompatibility in *Gillenia*

**Fig. 1:**
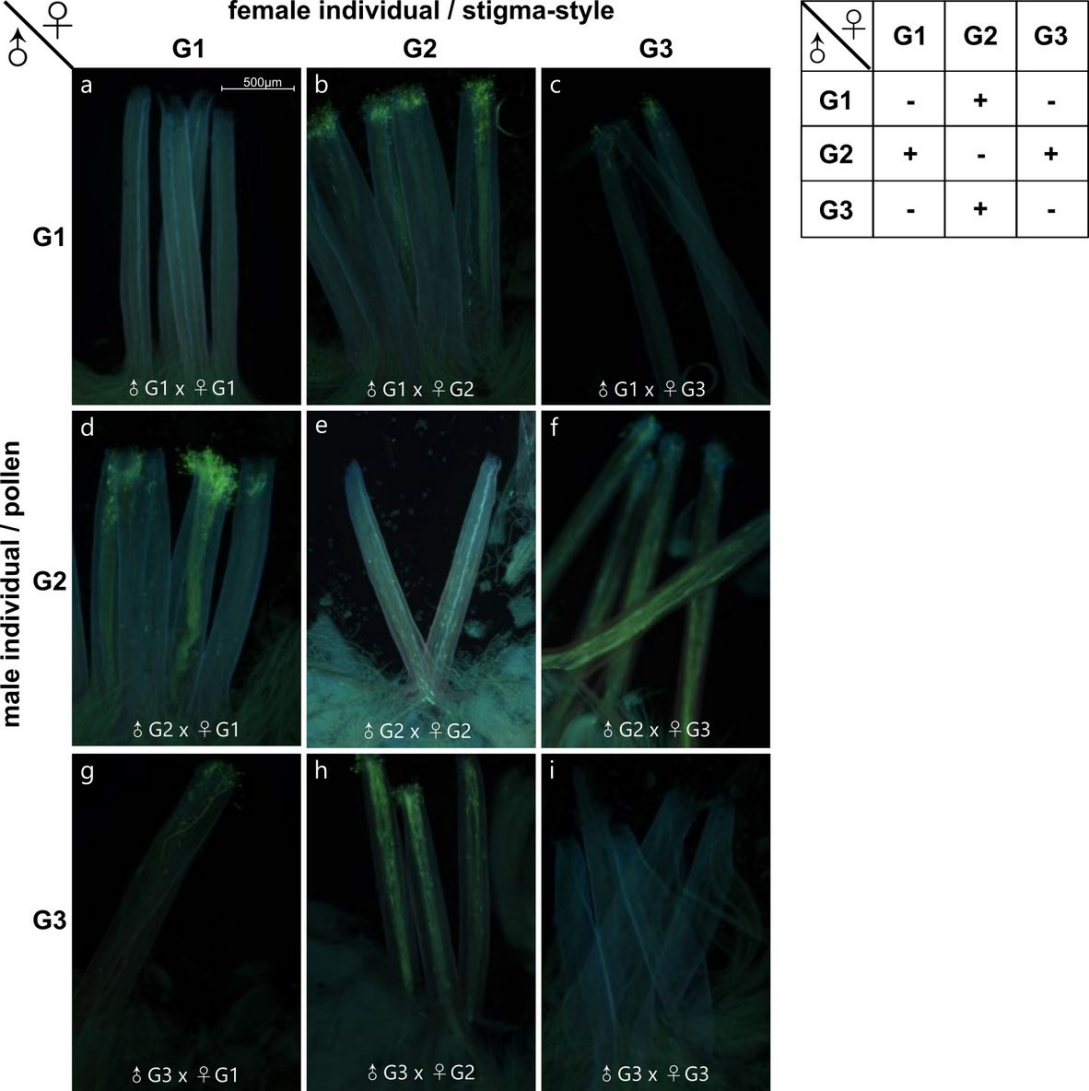
Self- and cross-(in)compatibility tested with pollen tube growth assays. Compound fluorescence images showing development of pollen tubes at 24 h after self- or cross-pollination of or between *Gillenia* individuals (‘G1’, ‘G2’, and ‘G3’). Pollen tube growth following self- (**a**,**e**,**i**) or reciprocal cross- pollination (**b**-**d**,**f**-**h**). Compatible reciprocal crosses were observed in a previous study^31^ between ‘G2’ and ‘G1’ (**b**,**d**) and ‘G2’ and ‘G3’ (**f**,**h**), while incompatible crosses were observed in a previous study in reciprocal crosses between ‘G1’ and ‘G3’ (**c**,**g**). Germinated pollen and pollen tubes are observed as green fluorescence. Scale bar, 500 μm. Analysis also performed at 48 h and showed reduced signal in all crosses (data not shown). Table shows summary of analysis from three replicates each with five styles; +, compatible; - incompatible.

In a previous study, facilitated self-pollination of *Gillenia* flowers failed to set fruit or seed over successive seasons suggesting *Gillenia* was self-incompatible^31^. Pollen tube growth assays were undertaken to explore self-incompatibility phenotype using hand pollination with self-pollen. It was found that all self-pollination assays resulted in no pollen tube growth confirming previous observations and presence of a self-incompatibility phenotype (Fig. 1a,e,i).

In the previous study, differential cross-compatibility phenotypes were observed between the three *Gillenia* individuals^31^. To determine reciprocal cross-incompatibility phenotype, pollen tube growth assays were undertaken after hand pollination with cross-pollen from different individuals. Complete pollen tube growth was observed in reciprocal crosses between individual ‘G2’ and individuals ‘G1’ or ‘G3’ (Fig. 1b,d,f,h), demonstrating cross-compatibility. However, limited or no pollen tube growth was observed in reciprocal crosses between individuals ‘G1’ and ‘G3’ (Fig. 1c,g) confirming previous observations and suggesting cross-incompatibility.

### A new highly contiguous genome for *Gillenia* reveals two putative *S*-loci

Due to their highly polymorphic nature, characterisation of *S*-loci requires highly contiguous genome assemblies. *Gillenia* version 1 (Gtr.v1) genome assembly had high synteny with selected Rosaceae genomes^31^ and comparison of Gtr.v1 with apple or peach (*Prunus persica*) genomes revealed syntenic regions in Gtr.v1 with their respective characterised *S*-loci. The apple *S*-locus, located on the distal end of apple linkage group 17^34^, was syntenic with the distal end of Gtr.v1 Chr03, while the *Prunus S*-locus, located on the distal end of peach linkage group 6^35^, was syntenic with the proximal end of Gtr.v1 Chr05 (Supplemental Fig. S1). Nucleotide sequence alignment between published Maleae *S-*RNase and *S*-locus F-Box Brother genes (see Methods) identified annotated gene models on Gtr.v1 Chr03 within the region syntenic with the apple *S*-locus, while alignment with published *Prunus S-*RNase genes identified regions only on unanchored contigs. This suggested Gtr.v1 lacked necessary contiguity for *S*-locus characterisation. To resolve this, a new *Gillenia* genome (Gtr.v2) was assembled using PacBio® Hi-Fi long-read sequencing (see Methods). High co-linearity was observed between Gtr.v1 and v2 genomes (Supplemental Fig. S2a,b) and BUSCO scores were comparable between versions. Gtr.v2 was haplotype-resolved with less unanchored scaffolds and almost no gaps (Table 1), illustrating higher contiguity.

**Table 1:**
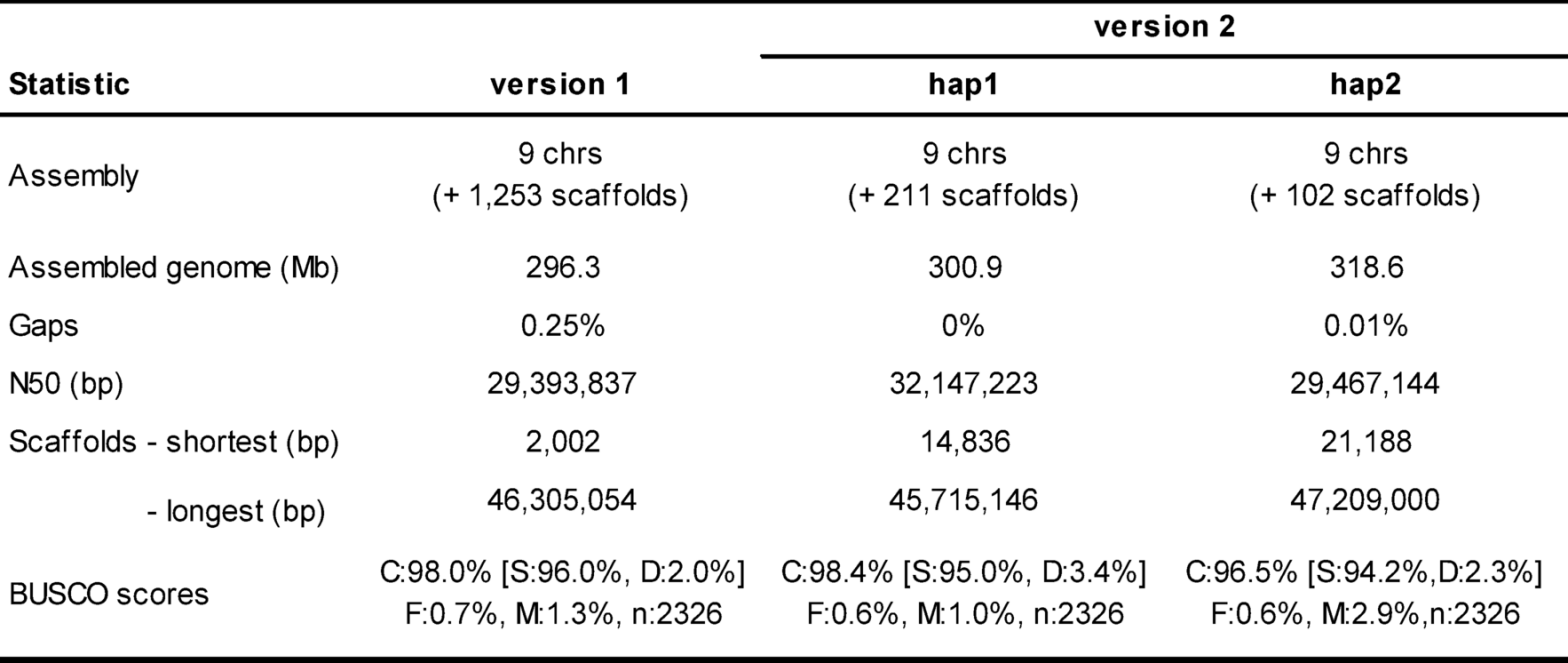
*Gillenia* v1 and v2 genome assembly metrics.

Nucleotide sequence alignment between Gtr.v2 and published Maleae and *Prunus S*-RNases, as described above, identified homology (Supplemental Fig. S2c), suggesting *Gillenia* possessed two putative *S*-loci: one on Chr03 orthologous to the Maleae *S*-locus (‘Mal *S*-locus’), and a second on Chr05 orthologous to the *Prunus S*-locus (‘Pru *S*-locus’). Automated and manual annotation identified putative *S*-genes within the *S*-loci on each haplotype. For simplicity haplotype 1 (gene model ID prefix ‘*Hap1*’) was used as reference for further analysis.

### Both Maleae and *Prunus*-like *S*-loci contain putative *S-*RNase genes

**Fig. 2:**
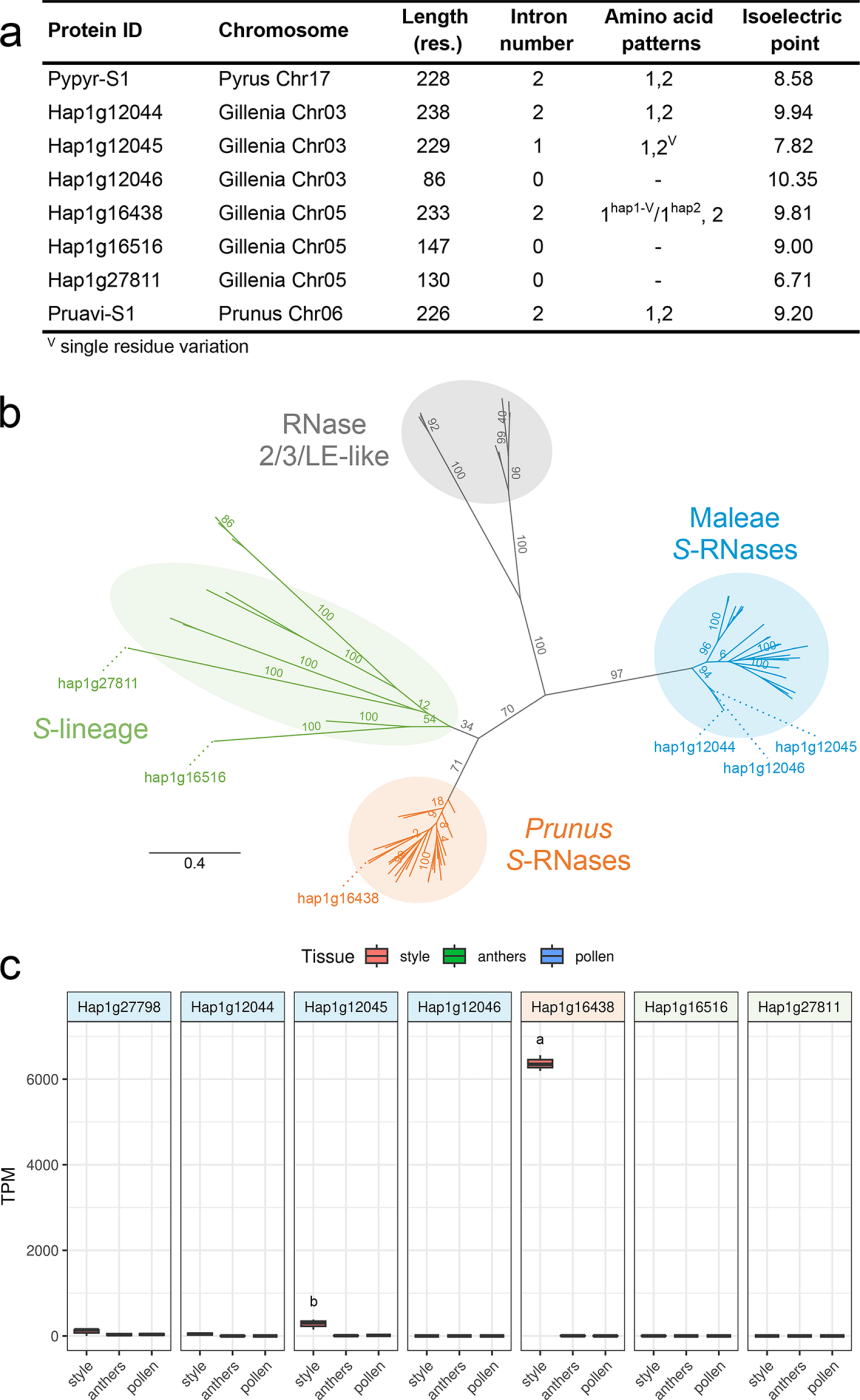
Molecular features of candidate *Gillenia S*-RNases. (**a**) Analysis of molecular signature of *S*-RNase genes in *Gillenia* candidates with two references (*Pyrus pyrifolia* Pypyr-S1 BAA32412.1 and *Prunus avium* Pruavi-S1 BAA83479.1). Protein sequence length (res., amino acid residues) shows truncation of some predicted proteins. Functional *S*-RNase genes have a maximum of two introns^36^, presence of amino acid patterns 1 and 2 and absence of amino acid pattern 4 (six residues ‘[CG]P[QLRSTIK][DGIKNPSTVY][ADEIMNPSTV][DGKNQST]’ occurring between pattern-2 and C3)^14^, and a basic isoelectric point (8 ≤ pI ≤ 10)^23^. pI calculated in Geneious Prime. (**b**) Phylogenetic analysis of *S*-RNase proteins from *Gillenia*, *Prunus*, and Maleae species. Clade nomenclature^23^ shows clades of ‘*Prunus S*-RNases’ (orange), ‘Maleae *S*-RNases’ (blue), a cluster containing ‘*S-*lineage 1’ and ‘*Malus S-*lineage 2’ clades with no known SI function (green) and outgroups of ‘RNase 2/3/LE-like’ genes (grey). Maximum likelihood bootstrap values from 100 datasets shown at branches. Scale is substitutions per site. (**c**) Expression of reference haplotype-1 *Gillenia S*-RNase genes in style/stigma, stamen (anther), and germinated pollen tissues, shown as transcripts per million (TPM), coloured banner of model ID indicates lineage corresponding to the phylogenetic tree in (**b**). Letters represent statistical significance groups from Tukey’s HSD test, all unlabelled data belong to the same group.

*S*-RNase-based GSI requires the activity of *S*-RNases belonging to Class III RNase T2 subfamily within the ribonuclease superfamily^4^. Within Class III, *S*-RNase with SI function can be distinguished from *S*-lineage RNases with no SI function in having a maximum of two introns^36^, presence of amino acid patterns 1 and 2 and absence of pattern 4^14^, and a basic isoelectric point (8 ≤ pI ≤ 10)^23^. Nucleotide sequence alignment between Gtr.v2 and published Maleae and *Prunus S*-RNases and further Conserved Domain Database search of putative loci (see Methods) identified seven candidate *S*-RNase genes: three on Chr05 in the ‘Pru *S*-locus’ and four on Chr03 in the ‘Mal *S*-locus’, however, one ‘Mal *S*-locus’ *S*-RNase (*Hap1g27798*) was putatively non-coding. The six protein-coding candidates were assessed for molecular features characteristic of *S*-RNases with SI function (Fig. 2). Only ‘Mal *S*-locus’ *Hap1g12044* and *Hap1g12045* and ‘Pru *S*-locus’ *Hap1g16438* encoded full-length proteins, had a maximum of two introns, encoded amino acid patterns 1 and 2, and were absent of *S*-lineage-specific amino acid pattern 4, and *Hap1g12044* and *Hap1g16438* had a predicted basic isoelectric point (Fig. 2a, Supplemental Fig. S3). *S*-RNase proteins have a characteristic secondary structure comprised of five conserved domains and hypervariable regions^25,37^. Analysis of secondary structure of the three full-length *S*-RNases showed, in addition to patterns 1 and 2 previously described, all three possessed the five conserved domains and the Rosaceae hypervariable domain (Supplemental Fig. S3). It was observed that the active site Lys residue in the C3 domain (K118 in Pruavi-S1, BAA83479), which is conserved in all *S*-RNases, was predicted to be mutated (K→T) in proteins of ‘Mal *S*-locus’ *Hap1g12044* and ‘Pru *S*-locus’ *Hap1g16438*, while the haplotype-2 allele of ‘Pru *S*-locus’ gene, *Hap2g17313*, did not have this mutation (Supplemental Fig. S3); a K→T mutation in the active site of C3 domain may alter *S*-RNase activity.

Phylogenetic analysis with published Maleae and *Prunus S*-RNase and *S*-lineage proteins showed that ‘Pru *S*-locus’ Hap1g16438 clustered in the ‘*Prunus S*-RNase’ clade, while the other two ‘Pru *S*-locus’ proteins clustered in the *S*-lineage clade which is not associated with SI function, and the three ‘Mal *S*-locus’ proteins clustered within the ‘Maleae *S*-RNases’ clade (Fig. 2b, Supplemental Fig. S4). *S*-RNase genes are characterised by high expression in the style and stigma^23,25^. Expression using mRNA-seq on style/stigma, stamen, and germinated pollen tissues was assessed for all seven putative *S*-RNase genes (Fig. 2c, Supplemental Fig. S5). The full-length *S*-RNases ‘Mal *S*-locus’ *Hap1g12044* and *Hap1g12045*, and ‘Pru *S*-locus’ *Hap1g16438*, were expressed in the style/stigma and not male reproductive tissues, but *Hap1g16438* expression was 30-fold higher in the style/stigma compared to the ‘Mal *S*-locus’ RNases. Of the two ‘Mal *S*-locus’ *S*-RNase genes, *Hap1g12045* had higher expression compared to *Hap1g12044*. Taken together, only the ‘Pru *S*-locus’ *Hap1g16438* had all molecular features consistent with a functional *S*-RNase, whereas the two full-length ‘Mal *S*-locus’ candidates had either insufficiently basic isoelectric point (*Hap1g12045*) or very low expression in style/stigma tissues (*Hap1g12044*).

### The ‘Pru *S*-locus’ *S*-RNase is highly polymorphic, has evolved under positive diversifying selection, and its alleles correlate with incompatibility phenotypes

*S*-genes are highly polymorphic through a combination of negative frequency-dependence, providing long-term maintenance of diverse alleles, and positive diversifying selection, facilitating molecular evolution of different recognition specificities^10,37^. To assess the degree of polymorphism amongst *Gillenia S*-RNases putative alleles were deduced from PacBio^®^ long-read IsoSeq and Illumina short-read transcriptomic datasets from three *Gillenia* individuals. For analysis of allelic diversity, ‘Pru *S*-locus’ *Hap1g16438* and ‘Mal *S*-locus’ *Hap1g12045* were selected based on RNA-seq expression levels (Fig. 2c). Amongst *Gillenia* individuals, four alleles were identified for ‘Pru *S*-locus’ *Hap1g16438* with divergent nucleotide (64.3–71.3%) and predicted protein (71.0–82.4%) identity. Four alleles were also identified for ‘Mal *S*-locus’ *Hap1g12045* and had greater conservation of nucleotide (99.3–99.9%) and predicted protein (98.3–99.6%) identity (Supplemental Fig. S6a-d). Additionally, single nucleotide polymorphism (SNP) density was calculated in a genome-wide context in the style/stigma transcriptomes of the three *Gillenia* individuals (Supplemental Fig. S6e) and showed increased SNP density at the ‘Pru *S*-locus’ compared to the ‘Mal *S*-locus’. Together, the data suggest only the ‘Pru *S*-locus’ *Hap1g16438* had sufficient polymorphic characteristics to control SI recognition specificity.

Cross-compatibility phenotypes were assessed for correlation with putative *S*-RNase alleles. Alleles of ‘Pru *S*-locus’ *Hap1g16438* were identified within each individual: G1 (alleles *pS1* (*Hap1g16438*), *pS2* (*Hap2g17313*)), G2 (alleles *pS3*, *pS4*), and G3 (allele *pS2*) (Supplemental Fig. S6f). Compatible reciprocal crosses were observed in the pollen tube growth assays between G2xG1 and G2xG3 (Fig. 1), suggesting the ‘Pru *S*-locus’ *S*-alleles correlated with these cross-compatibility phenotypes. Only one haplotype could be identified in G3 (*pS2*), and two alleles were identified in G1 (*pS1*, *pS2*), suggesting G1♂xG3♀ should yield a cross-compatibility phenotype while G3♂xG1♀ should have a cross-incompatibility. Pollen tube growth was not observed in either cross, suggesting additional male factors may be contributing to the cross-incompatibility phenotype of G1♂xG3♀. In contrast to the ‘Pru *S*-locus’ *S*-alleles, the ‘Mal *S*-locus’ alleles, in addition to the high sequence conservation (> 98.3% protein identity), did not correlate with incompatibility phenotypes (G1 (*mS1*, *mS2*), G2 (*mS1*, *mS3*), G3 (*mS1*, *mS4*)).

*S*-RNase genes display signatures of positive diversifying selection as nonsynonymous mutations change protein sequence and enable evolution of diverse recognition specificities^10,37^. Candidate *S-*RNase genes in *Gillenia* were therefore analysed for selective pressure using a branch-based phylogenetic approach (using CODEML) with *S*-RNases from eight species each from Maleae and *Prunus* (see Methods). Branch models assuming heterogeneous nonsynonymous/synonymous rate ratio (*ω* = *d*_N_/*d*_S_) for different branches of Maleae and *Prunus*-like *S*-RNase phylogenies were analysed (Table 2). In the *Prunus S-*RNase lineage, the alternate hypothesis (H1), which assumes the *Gillenia* branch has a different *ω* ratio to the *Prunus* branch, is rejected (2Δ*ℓ*< χ^2^_1,5%_) in favour of the null hypothesis (H0) of homogeneous *ω* ratio on all branches, suggesting the ‘Pru *S*-locus’ *S-*RNase in *Gillenia* is under similar selective pressure as known functional *Prunus S-*RNases. In comparison, in the Maleae *S-*RNase lineage, the null hypothesis of homogeneous *ω* ratio is rejected (2Δ*ℓ*> χ^2^_1,5%_) in favour of the alternate hypothesis that assumes the *Gillenia* branch has a different *ω* ratio to that in the Maleae branch, and correspondingly, a near 2-fold increase towards positive selection was evident in the Maleae branch comprised of known functional *S-*RNases, compared with the *Gillenia* branch. Branch-site models assuming heterogeneous *ω* across branches and amino acid residues (sites) were also analysed (Table 2). Compared to the *Prunus*-lineage *S*-RNases, the Maleae-lineage *S*-RNases observed a greater increase in proportions of 2a and 2b site classes (in which foreground branches (Maleae) are under positive selection while background branches (*Gillenia*) are under purifying selection or neutral evolution, respectively) between null and alternate hypotheses, and this change derived predominantly from a decrease in sites under neutral evolution (site class 1), suggesting the *Gillenia* ‘Mal *S*-locus’ *S*-RNase was under greater neutral selection compared to the *Gillenia* ‘Pru *S*-locus’ *S*-RNase, and supports branch model findings that the *Gillenia* ‘Pru *S*-locus’ *S*-RNase is under similar pressures to functional *Prunus S*-RNases.

**Table 2:**
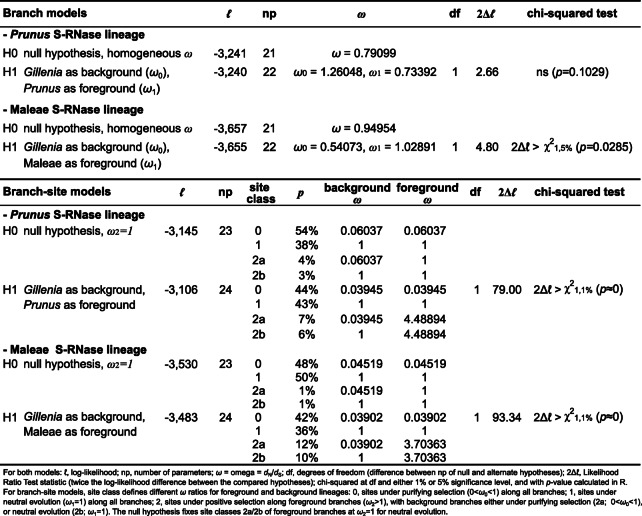
Likelihood ratio test (2Δ*ℓ*) and parameter estimates for positive selection of *Prunus*- and Maleae-lineage *S-RNases* under branch or branch-site models assuming heterogeneous *ω* across branches or branches and sites, respectively.

### Both Maleae and *Prunus*-like *S*-loci contain putative *S*-locus F-box genes

**Fig. 3:**
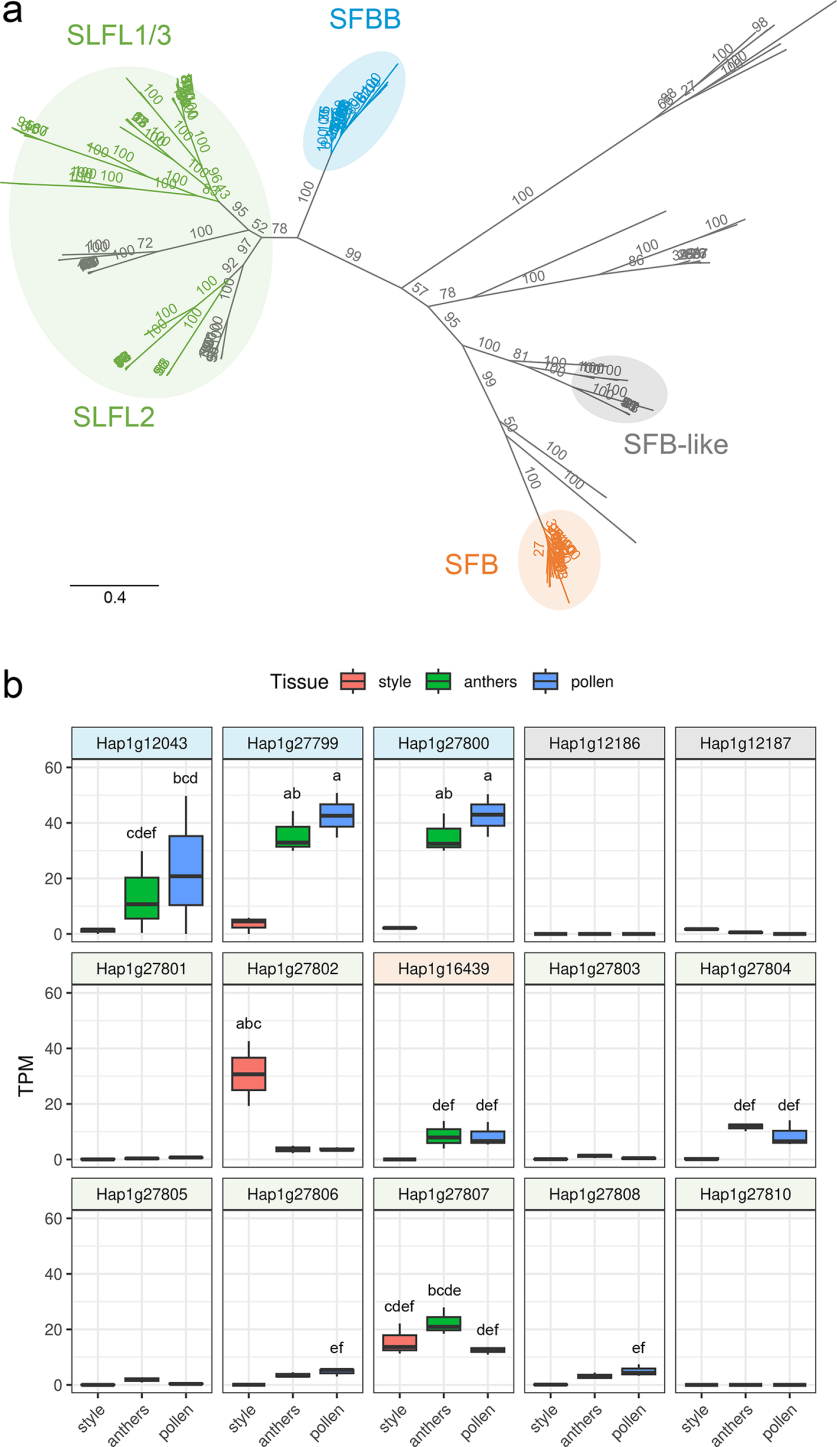
*Gillenia S*-locus F-boxes. (**a**) Phylogenetic analysis of *S*-locus F-box proteins from *Gillenia*, *Prunus*, and Maleae species. Clade nomenclature^23^ shows lineages of ‘*S*-locus F-Box Brothers’ (SFBB, blue), ‘*S*-Locus F-box Like’ (SLFL, green), ‘*S*-locus F-Box’ (SFB, orange), and ‘SFB-like’ (grey). Scale is substitutions per site. (**b**) Expression of reference haplotype-1 *Gillenia S*-locus F-box genes in style/stigma, stamen (anthers), and germinated pollen tissues, shown as transcripts per million (TPM), coloured banner of model ID indicates lineage corresponding to the phylogenetic tree in (**a**). Letters represent statistical significance groups from Tukey’s HSD test, unlabelled data belong to the ‘f’ group.

Nucleotide sequence alignment between Gtr.v2 and published Maleae *SFBB* and *Prunus* *SFB* genes followed by Conserved Domain Database search (see Methods) identified eight *S*-locus F-box genes on Chr03 ‘Mal *S*-locus’ and 16 on the Chr05 ‘Pru *S*-locus’. Phylogenetic analysis resolved the lineage of the predicted proteins (Fig. 3a; Supplemental Fig. S7). Of the eight F-boxes on the Chr03 ‘Mal *S*-locus’, three clustered with SFBB proteins, while four clustered with or near the SFB-like clade. Of the 16 F-boxes on Chr05 ‘Pru *S*-locus’, one clustered with SFB proteins, nine clustered with *S*-Locus F-box Like (SLFL) proteins, and the remaining six clustered in outgroups. *S*-locus F-box genes are typically highly expressed in pollen and stamen tissues^16,18^. mRNA-seq analysis of style/stigma, stamen, and germinated pollen tissues revealed that while all three *SFBB* genes and five *SLFL* genes have high expression in stamen and pollen tissues, low expression also occurred in style/stigma tissues. In contrast, the single *SFB* gene (*Hap1g16439*) was highly expressed in germinated pollen and stamen tissues and no expression was detected in the style/stigma (Fig. 3b; Supplemental Fig. S8). Analysis of protein structure of the pollen/stamen expressed *Gillenia S*-F-box candidates showed that each had the conserved F-box and variable domains specific to each lineage (Supplemental Fig. S9). Owing to a putatively recent segmental duplication at the ‘Mal *S*-locus’, two *SFBB* genes (*Hap1g27799* and *Hap1g27800*) were identical and the third *SFBB* gene (*Hap1g12043*) shared 93.7% nucleotide identity with *Hap1g27799* and *Hap1g27800*. Using mRNA-seq data, sequence diversity was found to be extremely low among the Maleae-lineage *Gillenia SFBB* genes (93.5–100% nucleotide identity), compared with *Prunus*-like *SFB* gene *Hap1g16439* (82.4–99.9% nucleotide identity) (Supplemental Fig. S10), supporting ‘Pru *S*-locus’ as a more highly polymorphic locus.

### Evolution of ‘Mal *S*-locus’ and ‘Pru *S*-locus’ in Rosaceae genomes

**Fig. 4:**
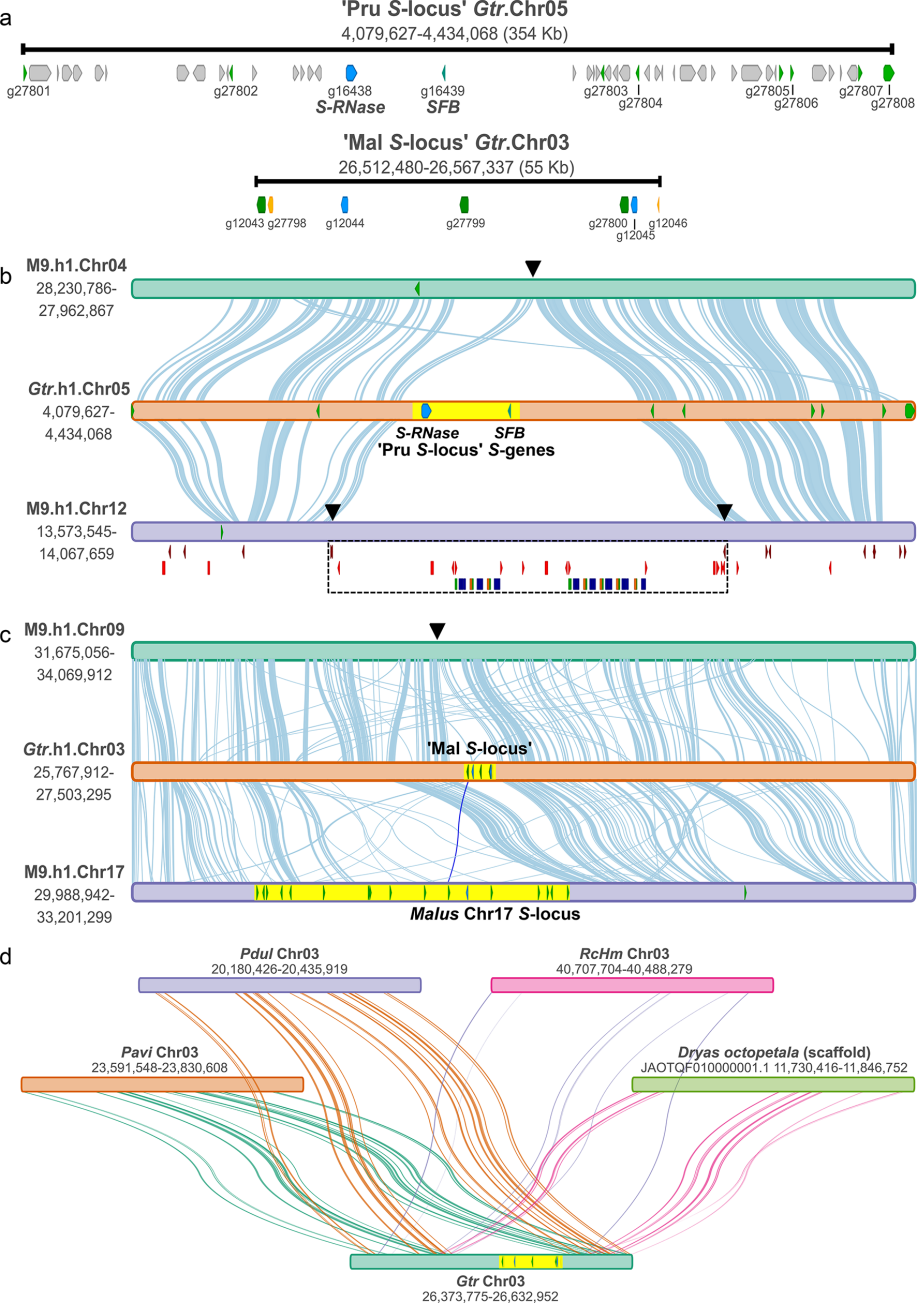
Evolution of the *Gillenia* ‘Mal and Pru *S*-loci’ in Rosaceae. (**a**) Schematic of the ‘Pru *S*-locus’ and ‘Mal *S*-locus’ in *Gillenia*. The ‘Pru *S*-locus’ on Gtr.Chr05 bears a single *S-RNase* (*hap1g16438*, blue triangle), single *SFB* (*hap1g16439*, turquoise triangle), and multiple *SLFL* genes (*hap1g27801-g27808*, green triangles), as well as genes with other functions (grey triangles). The ‘Mal *S*-locus’ on Gtr.Chr03 bears multiple *S-RNase* genes both full-length (*hap1g12044/hap1g12045*, blue triangles) and partial *S*-RNase genes (*hap1g27798/hap1g12046*, yellow triangles) and three *SFBB* genes (*hap1g12043/hap1g27799/hap1g27800*, green triangles). (**b**,**c**) Syntenic analysis between *Gillenia S*-loci with orthologous syntenic regions on *Malus domestica* ‘M9’ homoeologous chromosomes. *Gillenia S*-loci from (**a**) superimposed to scale onto Gtr chromosome segments. Yellow region denotes putative *S*-loci (i.e., core components (*S-RNase* and *SFB*) of the ‘Pru *S*-locus’, the ‘Mal *S*-locus’ on Gtr.Chr03, or the *Malus* Chr17 *S*-locus on ‘M9’ including *S-RNase* and *SFBB* genes and excluding a single outgroup F-box gene). ‘M9’ *S*-gene colour coding follows (**a**). M9.Chr12 insertion denoted by a dashed border, annotations below M9.Chr12 are (top-bottom), PIF/Harbinger transposons (dark red triangles), hAT transposons (light red triangles), and high-frequency domains identified within the insertion forming a repeating unit of photosynthesis (green) or ribosomal (brown) related domains with glycosyl hydrolase (dark blue) domains. Connecting lines denote synteny of minimum length 300 bp with greater than 78% identity, dark blue connecting line denotes microsynteny detected between *SFBB* genes Gtr.Chr03 *hap1g12043* and M9.Chr17. *SFBB3a* allele (MinLength = 1126 bp; 84.78%id). (**d**) Syntenic analysis between Gtr.Chr03 ‘Mal *S*-locus’ with orthologous syntenic regions from four species representing three Rosaceae subfamilies, *Prunus* (Amygdaloideae), *Rosa* (Rosoideae), *Dryas* (Dryadoideae). *Gillenia S*-loci from (**a**) superimposed to scale on Gtr.Chr03 segment and highlighted yellow. Connecting lines denote synteny of minimum length 120 bp with greater than 78% identity. Pavi, *Prunus avium* Tieton Genome v2.0^40^; Pdul, *Prunus dulcis* Nonpareil Genome v1.0^41^; RcHm, *Rosa chinensis* Homozygous Genome v2.0^42^; *Dryas octopetala* genome^43^. *Prunus* and *Rosa* genomes sourced from Genome Database for Rosaceae^44^, *Dryas* genome sourced from NCBI (www.ncbi.nlm.nih.gov).

Genomic architecture of the two putative *S*-loci in Gtr.v2 was characterised (Fig. 4a). The Chr05 ‘Pru *S*-locus’ in *Gillenia* contains the core components of SI regulation (*S*-RNase and *SFB*) and multiple *SLFL* genes, like the self-recognition *S*-loci observed in *Prunus* species^38^. In contrast, the Chr03 ‘Mal *S*-locus’ has more than one *S*-RNase, and few, near-identical *SFBB*-like genes, which is unlike the architecture of nonself-recognition *S*-loci observed in multiple species and comprised of a single highly polymorphic *S*-*RNase* gene with multiple *SFBB* genes^16,21^. As the Chr05 *S*-locus has the conserved architecture of functional *S*-loci, this suggests a *Prunus*-like self-recognition mechanism is regulating SI in *Gillenia*.

Comparative genomics was performed to understand putative origins and evolution of the ‘Mal *S*-locus’ and ‘Pru *S*-locus’ within apple. To assist this, a highly contiguous genome was constructed for the semi-vigorous apple (*M. domestica* Borkh) rootstock ‘M9’ using PacBio® HiFi long-read sequencing. This assembly achieved haplotype-level resolution, yielding 17 pseudomolecules per haplotype. Through scaffolding against the apple ‘GDDH13’ genome^39^, the pseudomolecules were built by 37 and 36 scaffolds for haplotypes 1 and 2, respectively. The order and orientation of scaffolds within each pseudomolecule per haplotype are shown in the AGP (A Golden Path) files (Supplemental Table S1). The resulting assembly showed an improved N50 of 38.4 Mb and 33.4 Mb for the two haplotypes (Supplemental Table S2). Moreover, 21 (constituting 95.04% of haplotype 1) and 29 (constituting 94.25% of haplotype 2) scaffolds were successfully anchored to the ‘M9’ genetic map (Supplemental Fig. S11), demonstrating synteny and anchoring. BUSCO scores of the ‘M9’ genome demonstrated high assembly quality (Supplemental Fig. S12).

Syntenic analysis performed between Chr05 ‘Pru *S*-locus’ and homoeologous *Malus* ‘M9’ Chr04/12 was conducted to explore putative *S*-locus evolution (Fig. 4b). The region containing the core SI components (*S-RNase* and *SFB*) was absent in both apple chromosomes and M9.Chr12 harboured additional non-syntenic sequence with M9.Chr04 suggesting an insertion. The putative M9.Chr12 insertion had PIF/Harbinger transposons at its borders, and within the insertion, hAT transposons bordered a repeating unit of ribosomal/photosynthesis-related and glycosyl hydrolase domains, suggesting a range of insertion mechanisms. One *SLFL*-like gene was identified on each *Malus* chromosome, sharing synteny with two different *Gillenia SLFL* genes, consistent with shared ancestry.

Syntenic analysis was conducted between Chr03 ‘Mal *S*-locus’ and homoeologous *Malus* ‘M9’ Chr09/17 to understand evolution of this putative *S*-locus (Fig. 4c). Consistent with evolutionary dynamics of *S*-loci, few syntenic links were identified between the ‘Mal *S*-locus’ and the apple orthologous chromosomes. However, microsynteny was detected between *SFBB*-like *Gillenia Hap1g12043* and *Malus SFBB3*a allele on M9.Chr17 (Fig. 4c; dark blue connector line), and between a transposable element in *Gillenia* and apple chromosomes (described below), suggesting independent evolution of the ‘Mal *S*-locus’ in the two species, but also putative shared ancestry of one *SFBB* gene.

To explore putative origins of the Chr03 ‘Mal *S*-locus’, an analysis of synteny between this region and identified syntenic regions from four genomes from species representing all three Rosaceae subfamilies (Rosoideae (*Rosa*), Amygdaloideae (*Prunus*), and Dryadoideae (*Dryas*)) was conducted. Despite syntenic regions either side of the *Gillenia *Chr03 'Mal* S*-locus’, no synteny was detected between the ‘Mal *S*-locus’ and syntenic regions from the four species (Fig. 4d), suggesting the Chr03 ‘Mal *S*-locus’ may have arisen independently in the Malodae ancestor.

### Features of *Gillenia* ‘Mal *S*-locus’ point to putative gene conversion mechanisms

**Fig. 5:**
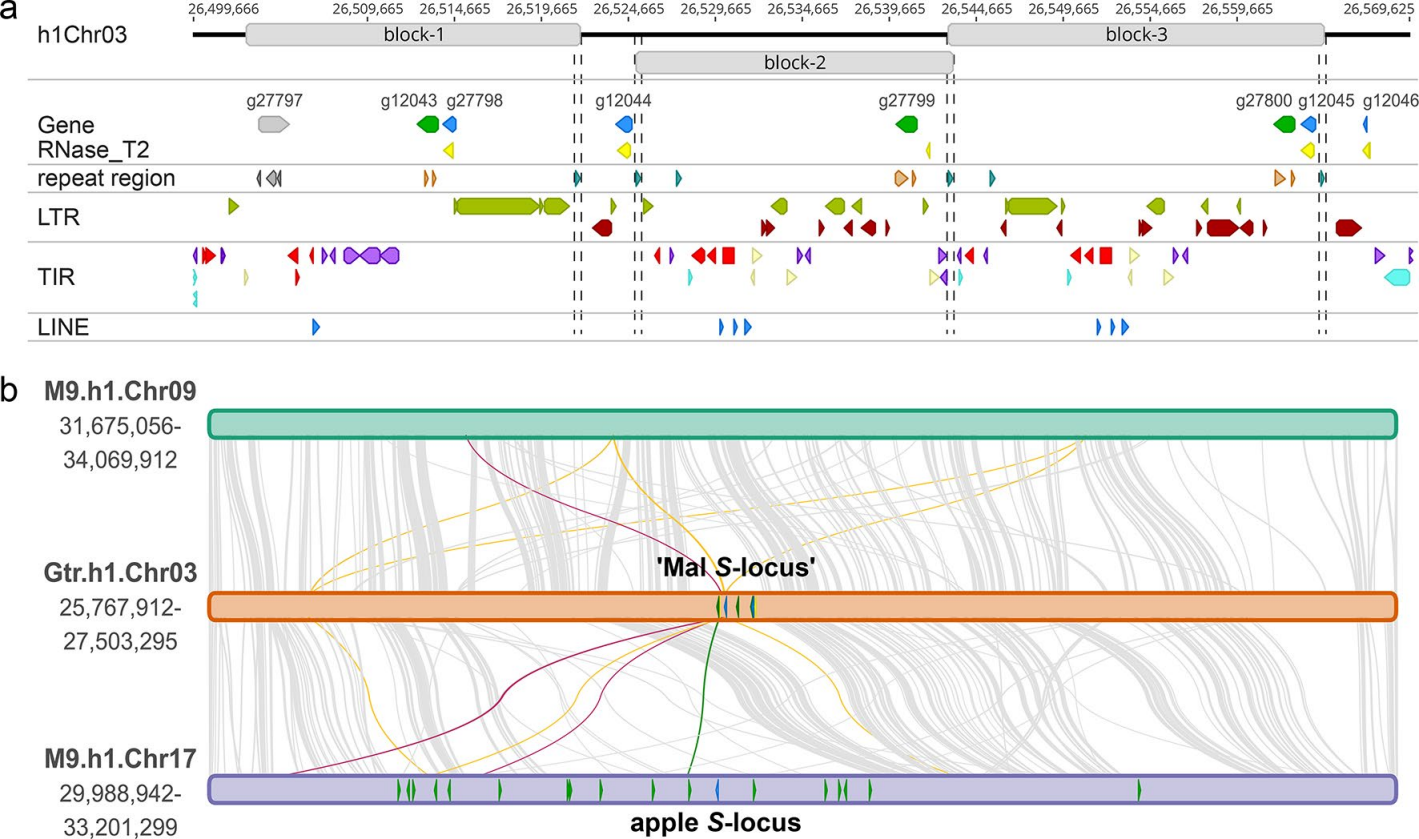
*Gillenia* ‘Mal *S*-locus’ has repeated blocks and repetitive sequences. (**a**) ‘Mal *S*-locus’ structure showing regions of putative segmental duplications with dashed lines indicating borders at repeat regions belonging to the same annotation family determined by the EDTA pipeline (teal triangles), found at the left and right borders of segmental blocks 2 and 3, and right border of segmental block 1. Top, grey boxes indicate regions of segmental duplications. Gene annotations are *SFBB* (green), full-length *S-RNase* genes (blue, *hap1g12044* and *hap1g12045*), partial *S-RNase* gene fragments (blue, *hap1g27798* and *hap1g12046*), and gene with no known *S*-locus function (grey, *hap1g27797*). RNase_T2 domains (yellow). Repeat regions (teal from the same family, orange from the same TE family, grey from additional and different TE families). LTR annotations are *Ty1/Copia* and *Ty3/Gypsy* (green), LTR retrotransposons (dark red). TIR annotations are hAT (light red), PIF/Harbinger (purple), Mutator (cream), CACTA (light blue). LINE elements (blue). (**b**) Syntenic analysis between *Gillenia* ‘Mal *S*-locus’ with orthologous syntenic regions on *Malus domestica* ‘M9’ homoeologous chromosomes, annotations follow Fig. 4c. Connecting lines denote synteny of minimum length 290 bp with greater than 78% identity. Connecting lines highlight syntenic relationships of interest: green, *Gillenia hap1g12043* and M9.Chr17 *SFBB3a* allele; maroon, *Gillenia* intact *Ty1/Copia* LTR with locations of the same TE family on both *Malus* chromosomes; light-orange, *Gillenia* annotated *Ty3/Gypsy* LTR with locations of the same family on both *Malus* chromosomes.

One hypothesis for the origin of the Maleae *S*-locus is through *de novo* evolution^23^ putatively arising through gene conversion^13^. Gene conversion can result from illegitimate recombination between non-homologous sequences or between duplicate sequences with high sequence similarity at ectopic and proximal locations, causing non-reciprocal (unidirectional) transfer of genetic information^45^. The Chr03 ‘Mal *S*-locus’ in *Gillenia* was assessed for features which may facilitate gene conversion (Fig. 5). Three segmental duplications, varying in size from 18.283 to 21.617 kb, were identified within the Chr03 ‘Mal *S*-locus’ which were bordered by highly similar sequences identified by the EDTA annotation pipeline^46^ as belonging to the same annotation family (Fig. 5a; Supplemental Fig. S13). Each segment included an *SFBB* gene positioned in tandem with an RNase_T2 domain-containing gene of either a full-length *S*-RNase (*hap1g12045*, block-3), non-coding partial *S*-RNase (*hap1g27798*, block-1), or remnant RNase_T2 domain only (block 2). Proximal positioning of highly similar sequence may permit illegitimate recombination and unidirectional transfer of genetic information.

Transposable elements (TEs) can accumulate in regions of low recombination such as *S*-loci^47,48^ and their presence does not imply causative effect in genome evolution; however, TE movements may propagate highly similar sequence to enable rare illegitimate recombination^49,50^. An intact *Ty1/Copia* LTR (identity 98.5%) positioned adjacent to *Gillenia SFBB hap1g12043* and encompassing non-coding partial *S*-RNase *hap1g27798* was found to be syntenic with three locations across *Malus* M9.Chr09 and M9.Chr17 (Fig. 5b, maroon lines) dispersed proximal to *Malus S*-locus *SFBB* genes (Fig. 5b, green line), suggesting both conservation and active replication proximal to other *SFBB* genes respectively before and after whole genome duplication in *Malus*. An annotated *Ty3/Gypsy* LTR also had synteny with TEs determined to be from the same family in multiple locations on both M9.Chr09 and M9.Chr17 (Fig. 5b, light orange lines), but with conservation of numbers suggesting no further replication after whole genome duplication. The intact *Ty1/Copia* LTR was also found to be actively transcribed in *Gillenia* with transcripts spanning the non-coding partial RNase_T2 *hap1g27798* gene (Supplemental Fig. S14). Presence of the intact *Ty1/Copia* LTR within the *Malus S*-locus may suggest a mechanism to propagate highly similar sequence throughout the locus.

## Discussion

In this study, we provide data which suggest a *Prunus*-like self-recognition mechanism governs SI in *Gillenia*. We have shown that *Gillenia* is a self-incompatible species and bears orthologous regions to Maleae nonself-recognition and *Prunus* self-recognition SI systems. However, only the ‘Pru *S*-locus’ had molecular features of a functional *S*-RNase including highest expression in the style/stigma tissues and evidence of positive diversifying selection, had highly polymorphic *S*-alleles, had *S*-RNase allele combinations which explain some SI phenotypes, and had conserved genome architecture relative to *Prunus S*-loci. The ‘Mal *S*-locus’ had more than one *S*-RNase, had fewer *S*-locus F-box genes necessary for a nonself-recognition mechanisms (typically greater than eight)^16,21^, *S*-genes lacked necessary degrees of polymorphism, evidence for positive selection of the *S*-RNase was weak, and *S*-RNase alleles did not correlate with cross-incompatibility phenotypes.

Some limitations of the data suggest the mechanism controlling SI regulation in *Gillenia* needs further analysis. A cross-incompatibility phenotype shown in the G1♂xG3♀ which may theoretically be cross-compatible, suggest other factors may be contributing to the incompatibility mechanism—this may suggest involvement of the Chr03 ‘Mal *S*-locus’. The function of the Chr03 ‘Mal *S*-locus’ remains to be elucidated, as genes present at the locus show sequence conservation and expression in reproductive organs. Two independent *S*-loci control self-incompatibility in *Fragaria*, but both loci are highly polymorphic^51^, with the Chr03 ‘Mal *S*-locus’ lacking this characteristic, a two-loci system does not seem feasible in *Gillenia*. Further work with a larger population and/or through segregation analyses would provide further clarity on the self-incompatibility mechanism in *Gillenia*.

Nonself-recognition systems are found in a wide range of species including species belonging to Solanaceae, Plantaginaceae, and Rosaceae families^9^ and are hypothesised to be the ancestral state^4^. A functional *Prunus*-like *S*-locus controlling self-incompatibility in *Gillenia* suggests the common ancestor of the Malodae supertribe (*Gillenia* + Maleae) also used a *Prunus*-like self-recognition mechanism. This may suggest evolution of widespread nonself-recognition systems is relatively labile. The data here suggest the ‘Pru *S*-locus’ may have been lost in the ancestor to the Maleae tribe but this requires further exploration of Maleae species beyond *Malus*. After putative loss, the Maleae nonself-recognition *S*-locus may have evolved independently from a rudimentary locus structure consisting of at least one *SFBB*, present in the Malodae ancestor, similar to the *Gillenia* Chr03 ‘Mal *S*-locus’. Evolution of the Maleae nonself-recognition system may have resulted from selective pressure to escape self-compatibility^8^. Self-compatibility may arise either, prior to polyploidisation, through loss of the self-recognition system^1^, or, after polyploidisation, through competitive interaction of diploid pollen^3,52^. Either hypothesis is possible, given the self-recognition system is absent on both apple Chr04/12 (supports deletion prior to polyploidisation), and apple Chr12 contains TEs bordering the insertion (supports TE movement induced by polyploidisation^50^. The Maleae *S*-locus putatively evolved *de novo* via gene conversion^13^. The data here show abundance of highly similar sequences throughout the Chr03 ‘Mal *S*-locus’. These sequences may facilitate illegitimate recombination and unidirectional transfer of genetic information, contributing to gene conversion.

Evidence of a *Prunus*-like *S*-locus controlling self-incompatibility in *Gillenia* suggests the common ancestor of the Amygdaloideae subfamily^32,33^ utilised a self-recognition system. In terms of the ancestral state of Rosaceae, only low support was found for an *SFB* gene on the *Dryas* (Dryadoideae subfamily) genome scaffold with high synteny to the ‘Pru *S*-locus’ (data not shown), but the low-quality assembly of the *Dryas* genome may contribute to this. However, a putative *SFB* gene was identified as an *S*-locus F-box gene in *Fragaria*^23^ (Rosoideae subfamily). Combined with the putatively dynamic nature of evolution of the Maleae nonself-recognition mechanism, it may be parsimonious to postulate that the Rosaceae ancestor bore an *SFB*-based self-recognition system, rather than a nonself-recognition system, as found in Maleae and *Rosa*^20^.

## Materials and methods

### Plant materials and growth conditions

Mature *Gillenia trifoliata* plants, purchased from Wake Robin Nursery, Balclutha, New Zealand (www.wakerobin.co.nz), were grown in glasshouse conditions as previously described^31^. Mature *Malus domestica* ‘M9’ (clone NZ9) plants, purchased from Waimea Nursery, Nelson, New Zealand (www.waimeanurseries.co.nz), were grown in research orchard conditions in Palmerston North, New Zealand.

### Genetic compatibility and pollen tube growth assays

Genetic self- and cross-compatibility studies in *Gillenia* were undertaken by hand-pollinating ten flowers per genotype using separate brushes with fresh pollen collected from flowers of the same (self-pollen) or different (cross-pollen) plant and performed in insect-free glasshouse conditions as previously described^31^. In vivo pollen tube growth assay^53^ was performed with modifications. Briefly, pistils were collected at 24 h and 48 h after pollination, fixed in FAA (37% formaldehyde/glacial acetic acid/50% ethanol, 5:5:90) and stored at 4 °C until use. Pistils were incubated in 4 N NaOH for 2 h to soften tissues, then washed thoroughly with water. Softened pistils were washed three times with 0.1% K_3_PO_4_, then incubated in 0.1% aniline blue in 0.1% K_3_PO_4_ for 24 h at 20 °C in darkness. Stained pistils were washed twice using 0.1% K_3_PO_4_ and gently squashed between a glass slide and cover slip in a drop of 0.1% aniline blue solution. Pollen tube growth in styles was observed with a compound fluorescence microscope (Nikon Eclipse Ni-E, Japan) with UV light at a wavelength of 365 nm. Three replicates per cross/self-pollination test were observed, with each replicate showing five styles.

In vitro pollen tube cultivation^54^ was performed with modifications. Mature pollen (BBCH stage 65^31^) was cultivated in 600 µL growth media (550 µM Ca(NO_3_)_2_, 1.6 mM H_3_BO_3_, 1.6 mM MgSO_4_, 1 mM KNO_3_, 440 mM sucrose, 5 mM 2-(*N*-morpholino) ethanesulfonic acid (MES), pH 6.5), and incubated in dark at 120 rpm at 25 °C for 5 h. Pollen tube growth was confirmed under the microscope. Germinated pollen grains were collected with centrifugation at 13,000 rpm, and the pelleted pollen was immediately processed for RNA extraction.

### Nucleic acid isolation, library construction and sequencing

For *Gillenia*, high molecular weight nuclear genomic DNA was extracted from isolated nuclei collected from young leaves as described previously^31^ and submitted to Leiden Genome Technology Center (Netherlands) for PacBio® HiFi library construction and sequencing.

For *Malus domestica* ‘M9’, young, fully expanded leaves (20 g) were collected and 2 µg of nuclear genomic DNA was extracted from isolated nuclei as previously described^55^, and used for PacBio® HiFi library construction and sequencing. A Single Molecule, Real-Time (SMRT) sequencing library (PacBio^®^) to obtain High Fidelity reads (HiFi) was generated by the service provider (Neogen^®^ Genomics, USA) and sequenced on the PacBio® Sequel II platform to generate subreads. Consensus reads (HiFi reads) were produced using the SMRT Link software of the PacBio analysis tool (https://github.com/caseywdunn/docker-smrtlink).

For RNA extraction, floral tissues at BBCH stages^31^ were immediately collected into lysis buffer, processed with a micro-homogeniser, and extracted with Spectrum Plant Total RNA Kit (Sigma-Aldrich, NZ). For PacBio^®^ long-read IsoSeq RNA-seq, a single composite library of pooled style/stigma and stamen tissues of open flowers (BBCH-65) from three individuals was prepared and sequenced on Sequel II by the Australian Genome Research Facility (AGRF, Australia). For short-read RNA-seq, RNA was extracted from three individuals, ten flowers each, from pre-opened (BBCH-60) style/stigma tissues, mature (BBCH-65) stamens, and germinated pollen grains prepared from mature (BBCH-65) flowers. mRNA-seq library construction and sequencing was performed by Novogene (Hong Kong) using the NovaSeq PE150 platform.

### Genome assembly

For the *Gillenia* genome, PacBio® Hi-Fi long-read DNA sequencing data were *de novo* assembled using hifiasm/v0.18.5-r500^56^. The resulting haplotype 1 and 2 contigs were processed in parallel and scaffolded into two sets of chromosome-scale assemblies using *Gillenia* genome v1^31^ as reference with homology-based assembler RagTag/v1.0.2^57^. Assembly statistics were calculated with Assemblathon^58^ and MUMmer/v3.23^59^. Telomeres were checked using telomeric-identifier (https://github.com/tolkit/telomeric-identifier). BUSCO/v5.2.2 analysis was used to verify assembly completeness^60,61^.

For the *Malus domestica* ‘M9’ genome, the haplotype-resolved genome was assembled using hifiasm/v0.18.5-r500^56^ in conjunction with PacBio® HiFi reads. Statistical evaluation of the resulting paired haplotypes was executed using Assemblathon^58^. The scaffolding was done with RagTag software^57^ using the whole genome alignment to the apple ‘Golden Delicious’ double haploid GDDH13v1.1 genome^39^ performed by nucmer software^59^ to produce an ‘agp’ file, which was then used by agptools (https://github.com/WarrenLab/agptools) to produce the two final haplotype assembly fasta files. The syntenic alignments (minimum alignment length of 18,000) between each haplotype and the reference genome were visualized through mummerplot R dot plots (https://jmonlong.github.io/Hippocamplus/2017/09/19/mummerplots-with-ggplot2/).

Additionally, the dual sets of haplotype-resolved scaffolds were anchored by aligning the 17 linkage groups established with a genetic map constructed for ‘M9’. This map was based on 646 and 626 SNPs markers per haplotype, respectively, which were extracted from the Illumina Infinium^®^ 20 K apple SNP array^62^ acquired from DNA of an ‘M9’ x ‘Robusta5’ crossing population^63^. The arrangement and alignment of scaffolds were performed in accordance with marker order from the linkage map using ALLMAPS/v0.7.7^59^. The final 17 pseudomolecules were merged into a single FASTA file per haplotype. Metrics of each haplotype were collected using Assemblathon^58^ and BUSCO values were calculated based on the eudicots_odb10 (2,326 genes) and embryophyte_odb10 (1,614 genes) datasets.

### Genome annotation

*De novo* repeats were deleted on Gtr.v2 using EDTA/v2^46,64^. The Gtr.v2 haplotyped genomes were soft masked using RepeatMasker/v4.0.5^65,66^. High quality PacBio long-read RNA IsoSeq data from pooled tissues (see ‘Nucleic acid isolation’ methods) was processed using pbmm2 (https://github.com/PacificBiosciences/pbmm2), with the haplotyped genomes indexed using ‘pbmm2 index’, followed by aligning IsoSeq reads with ‘pbmm2 align’. Illumina mRNA-Seq data were aligned to the soft-masked haplotyped genomes using STAR/v2.7.10a^67^. Alignments were fed into BRAKER/v2.1.5^68^ as evidence to predict gene models. The gene functional annotation was performed on the Mercator4 web server^69,70^ (https://www.plabipd.de/mercator_main.html).

Manual annotation was undertaken using alignment with short- and long-read RNA-seq data, Conserved Domain Database search using pfam00445 for *S-RNase* genes and pfam00646 and pfam01344 for *S*-locus F-box genes, homology identification using nucleotide BLAST (e-value < 1e-15) with published *S*-genes (Maleae *S*-RNases: U19793.1, AB002139.1, EF689008.1; *Prunus S*-RNases: AB028153.1, AB252415.1, AJ298314.1; Maleae SFBBs: AB270793.1, AB270796.1, AB270798.1; *Prunus* SFB/SLFLs: AB111518.1, AB092624.1, AB092627.1, AB092626.1), and whole chromosome alignment with characterised *S*-loci from *Pyrus* BAC clones AB545981 and AB545982^71^.

### Genome synteny analyses

Microsyntenic analysis used Synteny Viewer tool (Genome Database for Rosaceae^44^ to locate syntenic anchors followed by nucleotide alignment of regions containing syntenic anchors with progressiveMauve^72^ plugin in Geneious Prime/v2023.0.1 (Biomatters Ltd, Auckland, NZ) to explore genome annotation detail. Microsyntenic regions were analysed with dot plots generated using MUMmer/v3.23^59^ and visualised with NGenomeSyn^73^ with parameters described in text.

### *De Novo* transcriptome assembly

To analyse polymorphisms of *S*-gene candidates in *Gillenia*, nine short-read mRNA sequencing libraries from three tissue types (style/stigma, stamen, and germinated pollen) from three individuals (see Nucleic acid isolation methods above) were used for *de novo* transcriptome assembly using trinityrnaseq/v2.14.0^74^. Cleaned data were aligned to the haplotyped genomes and quantified using the nf-core/RNASeq pipeline^75^.

### Phylogenetic analysis

Putative *S*-RNase and *S*-F-box genes were identified *via* nucleotide BLAST search (e-value < 1e-15) with published *S*-genes for Maleae and *Prunus* (accession numbers same as used for manual annotation, see ‘Genome annotation’ methods section) and by Conserved Domain Database search of a 4 MB region encompassing each putative *S*-loci using pfam00445 for *S-*RNase genes and pfam00646 and pfam01344 for *S*-locus F-box genes. Phylogenetic analyses were conducted in Geneious Prime/v2023.0.1 (Biomatters Ltd, Auckland, NZ). Protein sequences for all putative *S*-genes were aligned using Clustal Omega/v1.2.2^76^. Regions of alignment were used to generate phylogenetic trees with PhyML/v3.3.20180621^77^ with JTT substitution model and bootstrap analysis of 100 datasets.

### Positive selection analyses

Protein and nucleotide sequences used for branch-wise comparisons were: *Prunus persica*-S1 AB252415.1, *P. dulcis*-Sj AAZ57491.1, *P. armeniaca*-S2 AAT69245.1, *P. mume*-S11 ABV71999.1, *P. salicina*-Se BAF91848.1, *P. cerasus*-S36a ABW17266.1, and *P. avium*-S22 ABR19609.1 for comparison with ‘Pru *S*-locus’ Hap1g16438 (*pS1* allele), and *M. domestica*-S27 AAB70516.1, *M. spectabilis*-S4 ADB85482.1, *Pyrus communis*-Sk BAE92269.1, *P. bretschneideri*-S34 ABD72921.1, *Sorbus aucuparia*-S18 ABP01658.1, *Cydonia oblonga*-S1 ATL75302.1, *Eriobotrya japonica*-S6, and *Crataegus pinnatifida*-S2 for comparison with ‘Mal *S*-locus’ Hap1g12045 (*mSa1* allele). Codon alignments were prepared from nucleotide sequences using Clustal Omega/v1.2.2^76^ and manually checked. Phylogenetic trees were prepared with RAxML^78^ using protein alignments prepared with Clustal Omega/v1.2.2^76^, and manually checked for known species relationships^32^. Ratios of non-synonymous substitutions to synonymous substitutions were calculated with codeml in PAML/v4.9i^79^, using default parameters.

## Electronic supplementary material

Below is the link to the electronic supplementary material.

Supplementary Material 1

## Data Availability

The datasets generated and/or analysed are available in the NCBI repository. For Gillenia genome, BioProjects PRJNA1196517 (haplotype 1, genome JBJYYC000000000) and PRJNA1197676 (haplotype 2, genome JBJYYD000000000) using BioSample SAMN45544832. For Malus genome, BioProjects PRJNA1196521 (haplotype 1, genome JBLJWW000000000) and PRJNA1197688 (haplotype 2, genome JBLJWX000000000) using BioSample SAMN45552643. For Gillenia IsoSeq, SRA SRR32173724 and SRR32173725 using BioSample SAMN45544833. For Gillenia mRNA-seq, SRA SRR32161566-SRR32161574 using BioSamples SAMN45544834-SAMN45544842. Also, the Gillenia genome and gene and TE annotations are available at the Genome Database for Rosaceae (www.rosaceae.org).
