# Supplementary material for: Characterisation of the Gillenia S-locus provides insight into evolution of the nonself-recognition self-incompatibility system in apple
Source: Sci Rep. 2025 Apr 26;15:14630. doi: 10.1038/s41598-025-99335-8 (PMC12033343; doi:10.1038/s41598-025-99335-8)
Supplement: Supplementary file 1 — Supplementary Material 1 [file 41598_2025_99335_MOESM1_ESM.pdf]

Supplementary Material

Table of Contents

Supplemental Tables **2**
Supplemental Figures **5**
References **20**

Table of Tables

Supplemental Table S1. 'A Golden Path' description of 'M9' assembly from scaffolds to chromosomes...... 2
Supplemental Table S2. *Malus domestica* 'M9' genome metrics. 4

Table of Figures

Supplemental Fig. S1. Mauve alignment between *Gtr*.v1 and *Malus* and *Prunus S*-loci 5
Supplemental Fig. S2. Syntenic analysis between *Gillenia* genomes v1 and v2. 6
Supplemental Fig. S3. Protein sequence alignment of full-length candidate *Gillenia S*-RNases. 7
Supplemental Fig. S4. Phylogenetic analysis of *S*-RNase proteins 8
Supplemental Fig. S5 Dot plots showing expression of *Gillenia S*-RNase genes. 9
Supplemental Fig. S6. Sequence diversity *Gillenia S*-RNase alleles and alignment of Pru *S*-locus alleles.... 10
Supplemental Fig. S7. Phylogenetic analysis of *S*-locus F-box proteins. 11
Supplemental Fig. S8. Dot plots showing expression of *Gillenia S*-locus F-box genes. 12
Supplemental Fig. S9. Protein sequence alignment of *Gillenia S*-locus F-box proteins. 13
Supplemental Fig. S10. Sequence diversity within *Gillenia S*-locus F-box alleles. 14
Supplemental Fig. S11. *Malus* **'**M9' genome synteny *versus* 'Golden Delicious' GDDHv1.1 genome......... 15
Supplemental Fig. S12. BUSCO scores for *Malus* 'M9' genome. 17
Supplemental Fig. S13. Details of the segmental duplications at *Gillenia* Chr03 'Mal *S*-locus' 18
Supplemental Fig. S14. Alignment of short RNA-seq and long IsoSeq reads at the LTR of interest 19

Supplemental Tables

Supplemental Table S1. 'A Golden Path' description of 'M9' assembly from scaffolds to chromosomes.

Object	Object beginning	Object end	Part number	Component type	Component ID or Gap length	Component beginning or Gap type	Component end or Linkage	Orientation or Linkage evidence
- Malus 'M9' Haplotype 1								
Chr01_RagTag	1	33644034	1	W	h1tg000005l	1	33644034	+
Chr02_RagTag	1	62149	1	W	h1tg000221l	1	62149	-
Chr02_RagTag	62150	62249	2	U	100	scaffold	yes	align_genus
Chr02_RagTag	62250	38780965	3	W	h1tg000001l	1	38718716	+
Chr02_RagTag	38780966	38781065	4	U	100	scaffold	yes	align_genus
Chr02_RagTag	38781066	38832502	5	W	h1tg000261l	1	51437	+
Chr03_RagTag	1	53850	1	W	h1tg000127l	1	53850	+
Chr03_RagTag	53851	53950	2	U	100	scaffold	yes	align_genus
Chr03_RagTag	53951	18289957	3	W	h1tg000021l	1	18236007	+
Chr03_RagTag	18289958	18290057	4	U	100	scaffold	yes	align_genus
Chr03_RagTag	18290058	38410948	5	W	h1tg000020l	1	20120891	-
Chr03_RagTag	38410949	38411048	6	U	100	scaffold	yes	align_genus
Chr03_RagTag	38411049	38432132	7	W	h1tg000252l	1	21084	+
Chr04_RagTag	1	19669437	1	W	h1tg000017l	1	19669437	+
Chr04_RagTag	19669438	19669537	2	U	100	scaffold	yes	align_genus
Chr04_RagTag	19669538	31990005	3	W	h1tg000018l	1	12320468	-
Chr05_RagTag	1	45919922	1	W	h1tg000007l	1	45919922	-
Chr06_RagTag	1	35783620	1	W	h1tg000015l	1	35783620	+
Chr07_RagTag	1	22012484	1	W	h1tg000016l	1	22012484	+
Chr07_RagTag	22012485	22012584	2	U	100	scaffold	yes	align_genus
Chr07_RagTag	22012585	23452024	3	W	h1tg000029l	1	1439440	+
Chr07_RagTag	23452025	23452124	4	U	100	scaffold	yes	align_genus
Chr07_RagTag	23452125	35780168	5	W	h1tg000011l	1	12328044	-
Chr08_RagTag	1	31961191	1	W	h1tg000004l	1	31961191	+
Chr09_RagTag	1	36027284	1	W	h1tg000008l	1	36027284	-
Chr10_RagTag	1	44016	1	W	h1tg000100l	1	44016	+
Chr10_RagTag	44017	44116	2	U	100	scaffold	yes	align_genus
Chr10_RagTag	44117	83667	3	W	h1tg000325l	1	39551	+
Chr10_RagTag	83668	83767	4	U	100	scaffold	yes	align_genus
Chr10_RagTag	83768	128759	5	W	h1tg000163l	1	44992	+
Chr10_RagTag	128760	128859	6	U	100	scaffold	yes	align_genus
Chr10_RagTag	128860	45122591	7	W	h1tg000003l	1	44993732	-
Chr11_RagTag	1	42504012	1	W	h1tg000010l	1	42504012	+
Chr12_RagTag	1	5128559	1	W	h1tg000025l	1	5128559	+
Chr12_RagTag	5128560	5128659	2	U	100	scaffold	yes	align_genus
Chr12_RagTag	5128660	9726231	3	W	h1tg000027l	1	4597572	+
Chr12_RagTag	9726232	9726331	4	U	100	scaffold	yes	align_genus
Chr12_RagTag	9726332	31657680	5	W	h1tg000012l	1	21931349	+
Chr12_RagTag	31657681	31657780	6	U	100	scaffold	yes	align_genus
Chr12_RagTag	31657781	32340457	7	W	h1tg000050l	1	682677	+
Chr13_RagTag	1	36196	1	W	h1tg000326l	1	36196	+
Chr13_RagTag	36197	36296	2	U	100	scaffold	yes	align_genus
Chr13_RagTag	36297	42343472	3	W	h1tg000013l	1	42307176	+
Chr14_RagTag	1	28363341	1	W	h1tg000002l	1	28363341	+
Chr14_RagTag	28363342	28363441	2	U	100	scaffold	yes	align_genus
Chr14_RagTag	28363442	28410159	3	W	h1tg000062l	1	46718	-
Chr14_RagTag	28410160	28410259	4	U	100	scaffold	yes	align_genus
Chr14_RagTag	28410260	30444080	5	W	h1tg000019l	1	2033821	-
Chr14_RagTag	30444081	30444180	6	U	100	scaffold	yes	align_genus
Chr14_RagTag	30444181	30642268	7	W	h1tg000048l	1	198088	-
Chr15_RagTag	1	56572007	1	W	h1tg000006l	1	56572007	+
Chr15_RagTag	56572008	56572107	2	U	100	scaffold	yes	align_genus
Chr15_RagTag	56572108	56605505	3	W	h1tg000172l	1	33398	+
Chr16_RagTag	1	40819692	1	W	h1tg000009l	1	40819692	+
Chr17_RagTag	1	34612713	1	W	h1tg000014l	1	34612713	-
Chr17_RagTag	34612714	34612813	2	U	100	scaffold	yes	align_genus
Chr17_RagTag	34612814	34657658	3	W	h1tg000096l	1	44845	+

Supplemental Table S1 cont. 'A Golden Path' description of 'M9' assembly from scaffolds to chromosomes.

Object	Object beginning	Object end	Part number	Component type	Component ID or Gap length	Component beginning or Gap type	Component end or Linkage	Orientation or Linkage evidence
**- Malus 'M9' Haplotype 2**								
Chr01_RagTag	1	24668096	1	W	h2tg000004l	1	24668096	+
Chr01_RagTag	24668097	24668196	2	U	100	scaffold	yes	align_genus
Chr01_RagTag	24668197	33650350	3	W	h2tg000023l	1	8982154	+
Chr02_RagTag	1	37768540	1	W	h2tg000001l	1	37768540	+
Chr03_RagTag	1	36878990	1	W	h2tg000015l	1	36878990	-
Chr04_RagTag	1	31329422	1	W	h2tg000002l	1	31329422	+
Chr05_RagTag	1	13702860	1	W	h2tg000013l	1	13702860	+
Chr05_RagTag	13702861	13702960	2	U	100	scaffold	yes	align_genus
Chr05_RagTag	13702961	47514284	3	W	h2tg000007l	1	33811324	-
Chr06_RagTag	1	35423565	1	W	h2tg000017l	1	35423565	-
Chr07_RagTag	1	3045911	1	W	h2tg000030l	1	3045911	+
Chr07_RagTag	3045912	3046011	2	U	100	scaffold	yes	align_genus
Chr07_RagTag	3046012	36533149	3	W	h2tg000014l	1	33487138	-
Chr08_RagTag	1	31018377	1	W	h2tg000008l	1	31018377	+
Chr09_RagTag	1	54370	1	W	h2tg000072l	1	54370	-
Chr09_RagTag	54371	54470	2	U	100	scaffold	yes	align_genus
Chr09_RagTag	54471	35036718	3	W	h2tg000022l	1	34982248	+
Chr10_RagTag	1	43271908	1	W	h2tg000005l	1	43271908	+
Chr11_RagTag	1	39749445	1	W	h2tg000010l	1	39749445	-
Chr11_RagTag	39749446	39749545	2	U	100	scaffold	yes	align_genus
Chr11_RagTag	39749546	41932324	3	W	h2tg000025l	1	2182779	-
Chr12_RagTag	1	12155010	1	W	h2tg000021l	1	12155010	+
Chr12_RagTag	12155011	12155110	2	U	100	scaffold	yes	align_genus
Chr12_RagTag	12155111	18820139	3	W	h2tg000012l	1	6665029	+
Chr12_RagTag	18820140	18820239	4	U	100	scaffold	yes	align_genus
Chr12_RagTag	18820240	20481807	5	W	h2tg000028l	1	1661568	+
Chr12_RagTag	20481808	20481907	6	U	100	scaffold	yes	align_genus
Chr12_RagTag	20481908	27566056	7	W	h2tg000024l	1	7084149	-
Chr12_RagTag	27566057	27566156	8	U	100	scaffold	yes	align_genus
Chr12_RagTag	27566157	31619246	9	W	h2tg000026l	1	4053090	+
Chr13_RagTag	1	31113885	1	W	h2tg000003l	1	31113885	-
Chr13_RagTag	31113886	31113985	2	U	100	scaffold	yes	align_genus
Chr13_RagTag	31113986	31152369	3	W	h2tg000077l	1	38384	-
Chr13_RagTag	31152370	31152469	4	U	100	scaffold	yes	align_genus
Chr13_RagTag	31152470	43480823	5	W	h2tg000006l	1	12328354	+
Chr14_RagTag	1	43541	1	W	h2tg000111l	1	43541	-
Chr14_RagTag	43542	43641	2	U	100	scaffold	yes	align_genus
Chr14_RagTag	43642	32509739	3	W	h2tg000016l	1	32466098	+
Chr15_RagTag	1	2061862	1	W	h2tg000032l	1	2061862	-
Chr15_RagTag	2061863	2061962	2	U	100	scaffold	yes	align_genus
Chr15_RagTag	2061963	2213201	3	W	h2tg000035l	1	151239	+
Chr15_RagTag	2213202	2213301	4	U	100	scaffold	yes	align_genus
Chr15_RagTag	2213302	2877498	5	W	h2tg000050l	1	664197	-
Chr15_RagTag	2877499	2877598	6	U	100	scaffold	yes	align_genus
Chr15_RagTag	2877599	3508395	7	W	h2tg000034l	1	630797	-
Chr15_RagTag	3508396	3508495	8	U	100	scaffold	yes	align_genus
Chr15_RagTag	3508496	3560254	9	W	h2tg000041l	1	51759	+
Chr15_RagTag	3560255	3560354	10	U	100	scaffold	yes	align_genus
Chr15_RagTag	3560355	55114971	11	W	h2tg000019l	1	51554617	-
Chr16_RagTag	1	27369701	1	W	h2tg000011l	1	27369701	+
Chr16_RagTag	27369702	27369801	2	U	100	scaffold	yes	align_genus
Chr16_RagTag	27369802	40296101	3	W	h2tg000018l	1	12926300	+
Chr17_RagTag	1	12722238	1	W	h2tg000009l	1	12722238	+
Chr17_RagTag	12722239	12722338	2	U	100	scaffold	yes	align_genus
Chr17_RagTag	12722339	33249712	3	W	h2tg000027l	1	20527374	+

Scaffolding against apple reference genome of 'Golden Delicious' (double haploid GDDH13v1.1)[2].

Supplemental Table S2. *Malus domestica* 'M9' genome metrics.

	'M9' hifiasm assembly		'M9' assembly after anchoring to 'M9' genetic map		'M9' assembly after scaffolding against GDDH13v1.1 genome	
**Assembly metrics**	**Hifiasm hap1**	**Hifiasm hap2**	**After anchoring hap1**	**After anchoring hap2**	**After scaffolding vs ref hap1**	**After scaffolding vs ref hap2**
**Number of pseudomolecules**	_	_	17	17	17	17
**Number of scaffolds**	327	115	21	29	37	36
**Total size fo scaffolds (Mb)**	669	655	636	618	653	647
**Longest scaffold (Mp)**	56.5	51.6	56.5	52.1	56.6	51.5
**Shortest scaffold (Mb)**	0.17	0.21	22	27	0.21	0.38
**Number of scaffolds > 1 Mb**	24	30	21	29	24	29
**Number of scaffolds > 200 Kb**	31	41	21	29	25	31
**N50 scaffold length (Mb)**	36	33	38	35	38.4	33.4
**Busco values embryophyta (%)**	C:98.9 [S:63, D:35.9] F:0.6, M:0.5	C:98.7 [S:63.2, D:35.5] F:0.6, M:0.7	C:96.3 [S:63.8, D:32.5] F:0.9, M:2.8	C:98.8 [S:63.3, D:35.5] F:0.6, M:0.6	C:98.8 [S:63.1, D:35.7] F:0.6, M:0.6	C:98.7 [D:63.2, S:35.5] F:0.6, M:0.7
**Busco values euditcots (%)**	C:98.7 [S:60, D:38.7] F:0.5, M:0.8	C:98.4 [S:60.4, D:38] F:0.6, M:1	C:96.4 [S:61, D:35.4] F:0.6, M:3	C:98.2 [S:60.1, D:38.1] F:0.5, M:1.3	C:98.5 [S:59.9, D:38.6] F:0.5, M:1	C:98.2 [D:60.1, D:38.1] F:0.6, M:1.2

Supplemental Figures

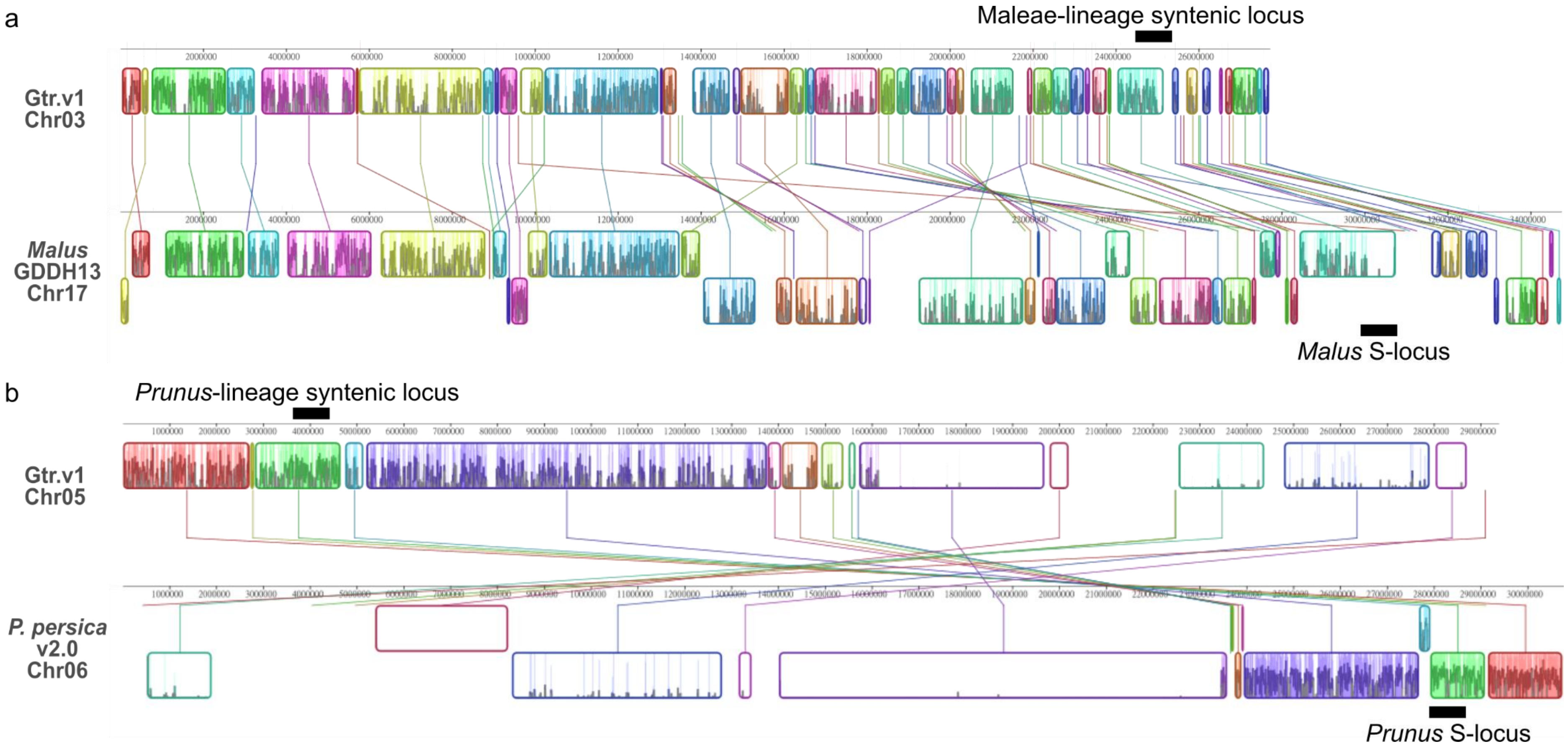

Supplemental Fig. S1. Mauve alignment between *Gtr*.v1 and *Malus* and *Prunus S*-loci

Progressive Mauve alignment between syntenic chromosomes of *Gillenia* genome v1 (Gtr.v1)[3] with chromosomes bearing S-loci from (a) *Malus domestica* GDDH13[2] and (b) *Prunus persica* genome v2.0[4].

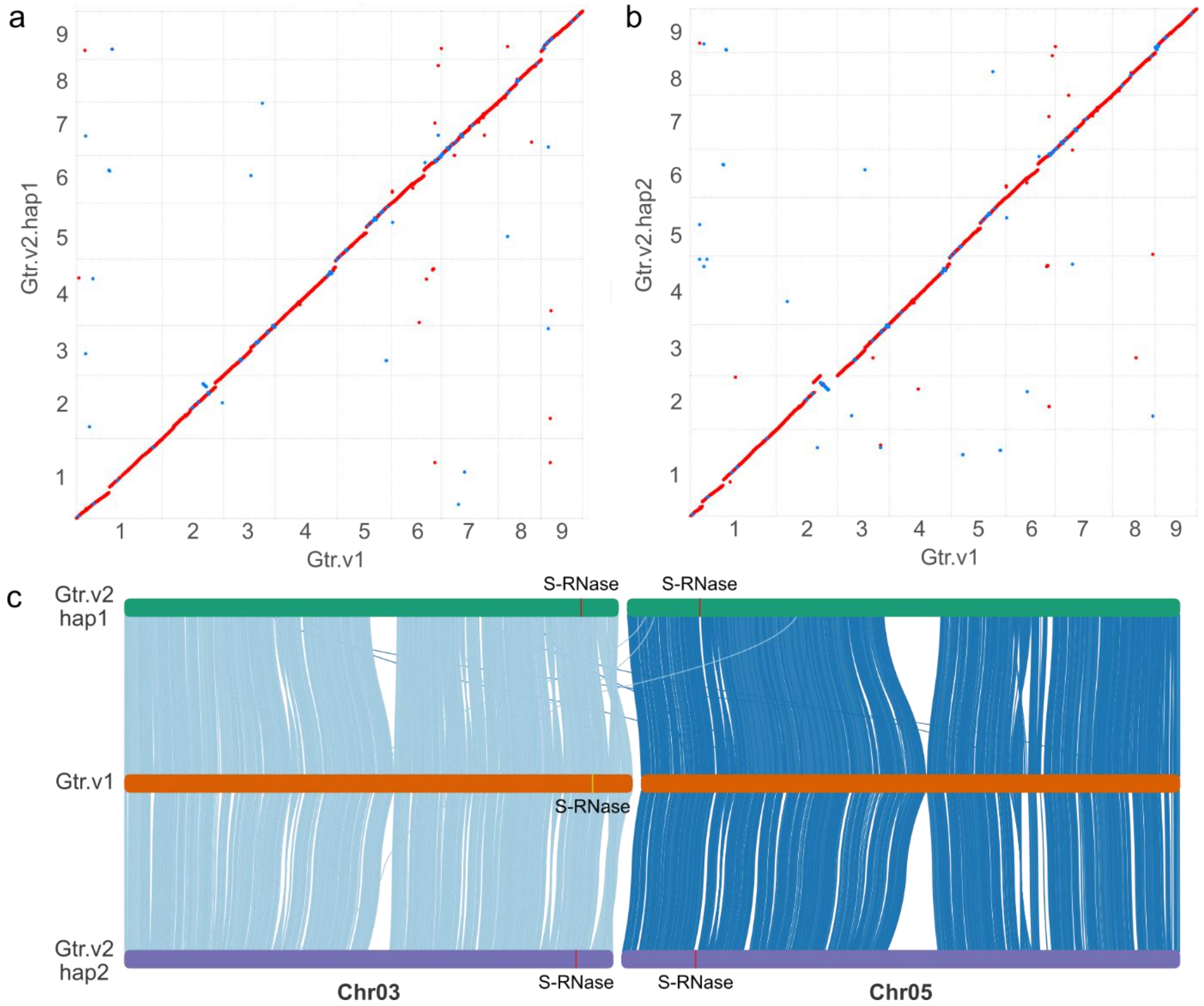

Supplemental Fig. S2. Syntenic analysis between *Gillenia* genomes v1 and v2.

(a,b) Dot plot analysis of *Gillenia* whole genome *Gtr*.v1 with either *Gtr*.v2 haplotype 1 (hap 1; a) or haplotype 2 (hap 2; b). Each dot represents a collinear genomic block with a 95% nucleotide identity over a 10 kb in length. Red and blue represent forward and reverse matches, respectively. (C) Syntenic analysis of Chr03 and Chr05 between *Gtr*.v1 (orange) and *Gtr*.v2 hap1 (green) or *Gtr*.v2 hap2 (purple). The location of putative *Gillenia S*-RNase genes showing homology to published Maleae and *Prunus S*-RNase genes on Chr03 and Chr05 (where identified) is indicated on each chromosome.

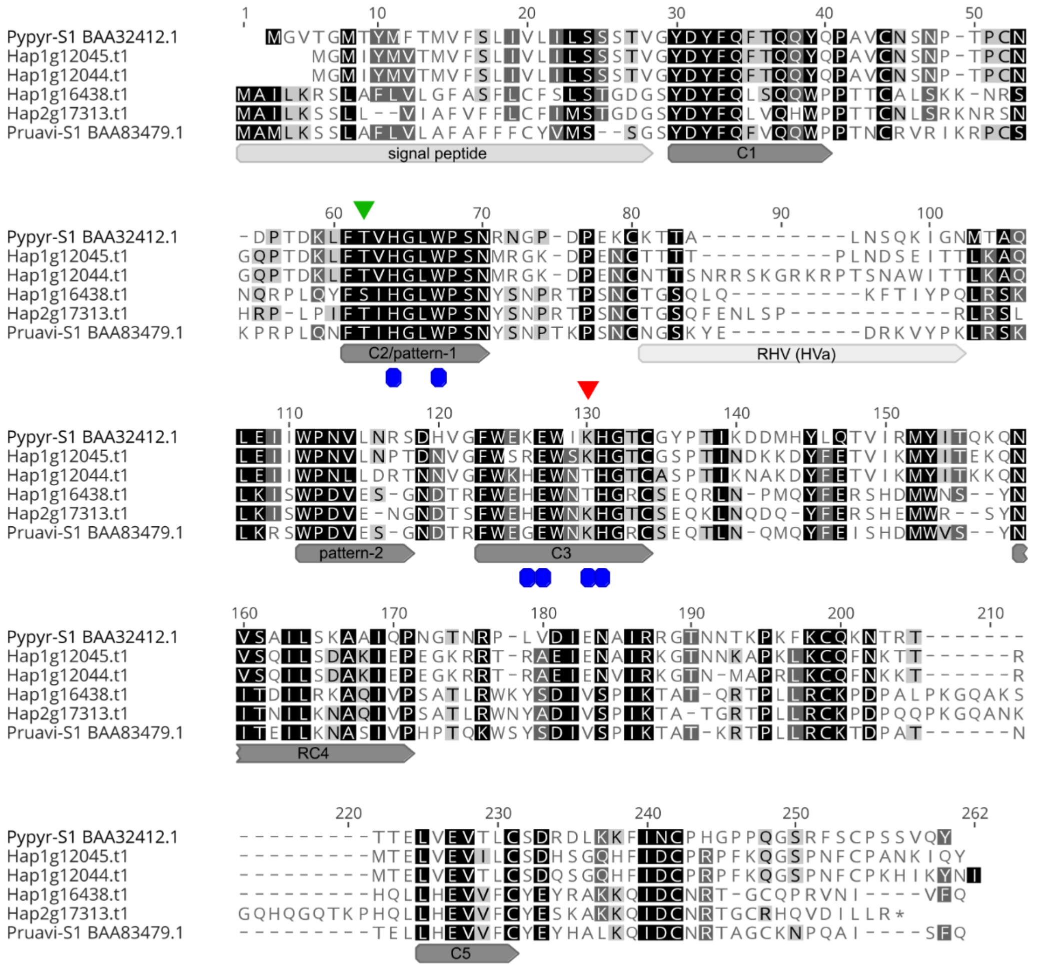

Supplemental Fig. S3. Protein sequence alignment of full-length candidate *Gillenia S*-RNases.

Protein sequence alignment of full-length candidate *S*-RNase genes with reference genes from *Pyrus pyrifolia* (Pypyr-S1) and *Prunus avium* (Pruavi-S1). Conserved domains (C1-C5), pattern-1 = C2, pattern-2, variable domains (RHV (HVa) and active sites (blue rectangles) are depicted. Pattern-4 specific to *S*-lineage proteins (six residues '[CG]P[QLRSTIK][DGIKNPSTVY][ADEIMNPSTV][DGKNQST]' would occur between pattern-2 and C3). One residue variation was identified in one allele of the 'Pru *S*-locus' Hap1g16438 (green triangle). Conserved Pruavi-S1 residue K118 (alignment residue 130) is predicted to be mutated K→T in *Gillenia S*-RNases 'Mal *S*-locus' Hap1g12044 and in the 'Pru *S*-locus' Hap1g16438 allele – the haplotype-2 'Pru *S*-locus' *S*-RNase allele, Hap2g17313, is however not mutated (red triangle).

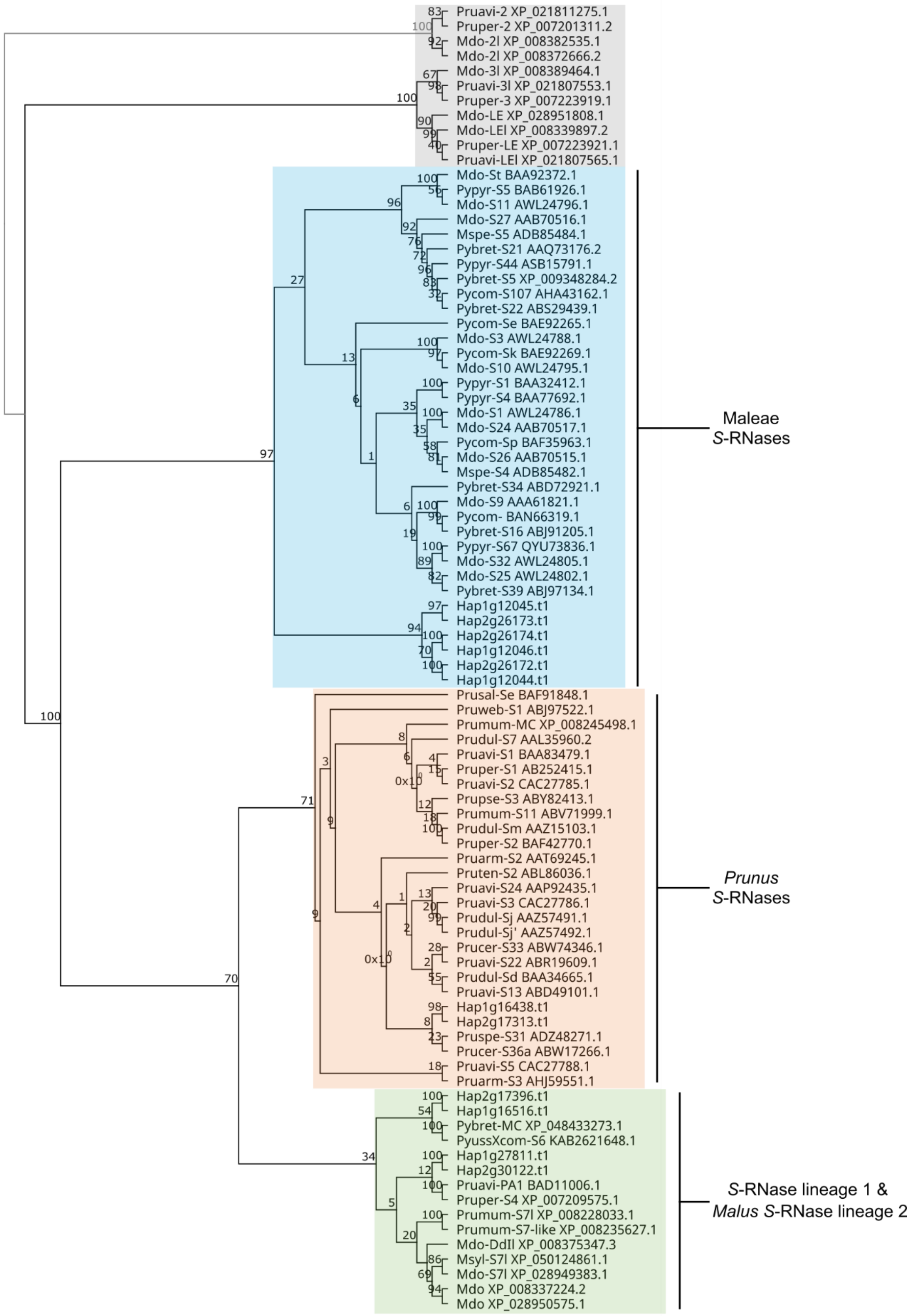

Supplemental Fig. S4. Phylogenetic analysis of *S*-RNase proteins

Phylogenetic analysis of *S*-RNase proteins from *Gillenia*, *Prunus*, and Maleae species. Clade nomenclature follows[1] and shows lineages of '*Prunus S*-RNases' (orange), 'Maleae *S*-RNases' (blue), a cluster containing both '*S-RNase* lineage 1' and '*Malus S-RNase* lineage 2' (green) and outgroups (grey). Maximum likelihood bootstrap values from 100 datasets shown at branches. NCBI codes for *Prunus* and Maleae proteins, *Gtr*.v2 gene model IDs for *Gillenia* proteins.

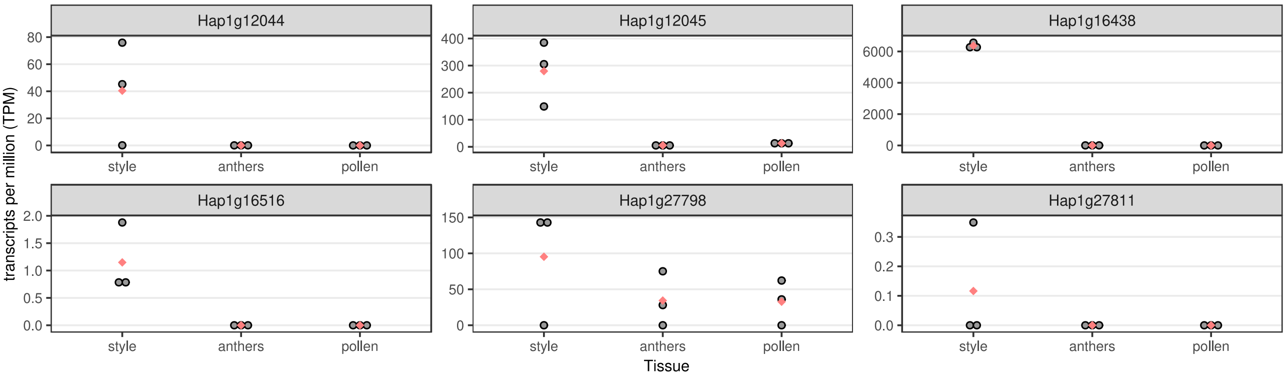

Supplemental Fig. S5 Dot plots showing expression of *Gillenia S*-RNase genes.

Gene expression in style/stigma, stamen (anthers), and germinated pollen, shown as transcripts per million (TPM), grey circles represent replicates, pink circles represent mean. *Malus S*-RNase genes are *Hap1g12044* and *Hap1g12045*; *Prunus S*-RNase gene is *Hap1g16438*; *Hap1g16516* and *Hap1g27811* belong to '*S-RNase* lineage 1' and '*Malus S-RNase* lineage 2' clade; *Hap1g27798* is a putative non-coding gene on 'Mal S-locus'.

a

	Gtr_pS4	Gtr_pS1	Gtr_pS3	Gtr_pS2
Gtr_pS4		67.5%	67.9%	64.3%
Gtr_pS1	67.5%		71.3%	68.1%
Gtr_pS3	67.9%	71.3%		86.8%
Gtr_pS2	64.3%	68.1%	86.8%	

c

	Gtr_mSa3	Gtr_mSa2	Gtr_mSa1	Gtr_mSa4
Gtr_mSa3		99.3%	99.6%	99.4%
Gtr_mSa2	99.3%		99.7%	99.6%
Gtr_mSa1	99.6%	99.7%		99.9%
Gtr_mSa4	99.4%	99.6%	99.9%	

b

	Gtr_pS2	Gtr_pS3	Gtr_pS1	Gtr_pS4
Gtr_pS2		82.4%	73.4%	74.4%
Gtr_pS3	82.4%		71.0%	71.9%
Gtr_pS1	73.4%	71.0%		76.1%
Gtr_pS4	74.4%	71.9%	76.1%	

d

	Gtr_mSa3	Gtr_mSa4	Gtr_mSa1	Gtr_mSa2
Gtr_mSa3		98.3%	98.7%	98.3%
Gtr_mSa4	98.3%		99.6%	99.1%
Gtr_mSa1	98.7%	99.6%		99.6%
Gtr_mSa2	98.3%	99.1%	99.6%	

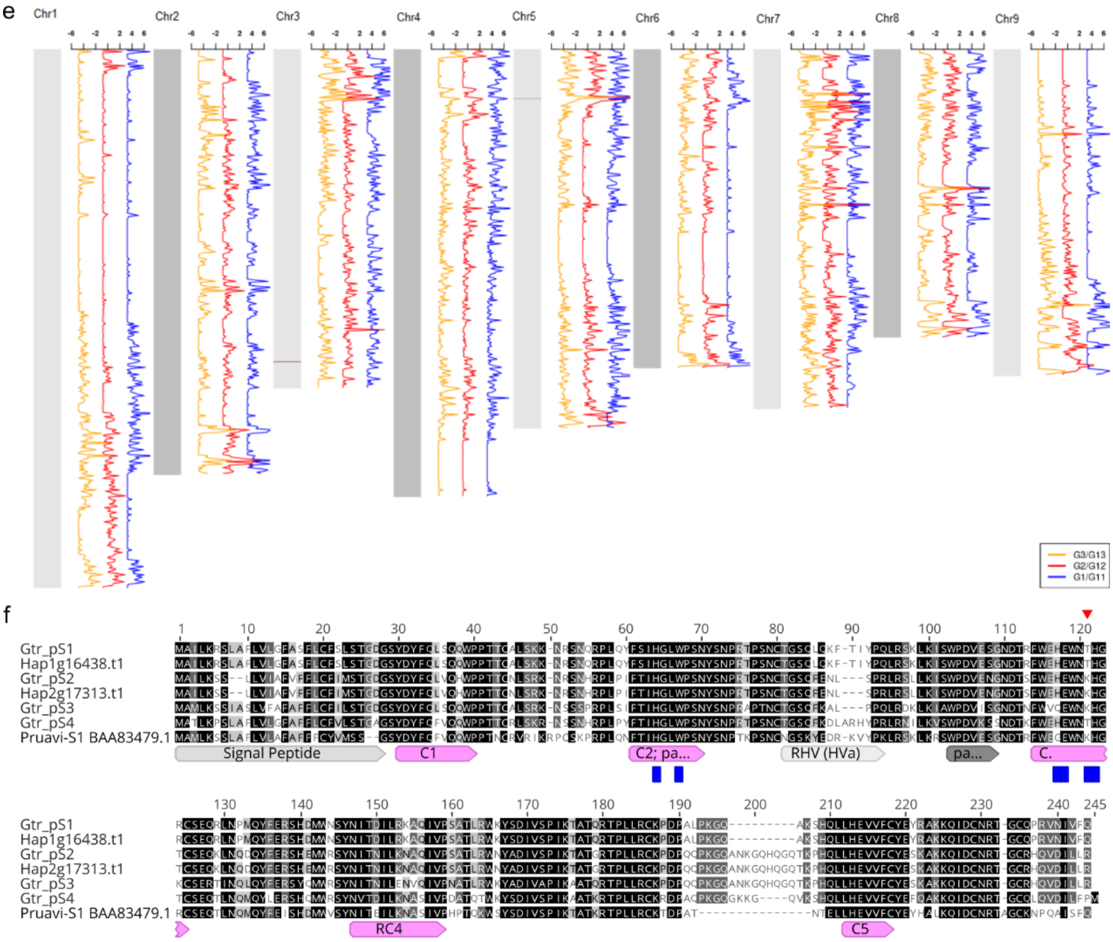

Supplemental Fig. S6. Sequence diversity *Gillenia S*-RNase alleles and alignment of Pru *S*-locus alleles.

(a-d) Allelic distance matrices. Nucleotide (a,c) and protein (b,d) distance matrices showing percent identity for the putative functional *S*-RNase alleles of the ‘Pru *S*-locus’ gene *hap1g16438.t1* (a,b) and ‘Mal *S*-locus’ gene *hap1g12045.t1* (c,d). (e) Whole genome overview plot of single nucleotide polymorphism (SNP) densities along hap1 chromosomes detected in the style/stigma transcriptomes of three *Gillenia* individuals (G1-G3). *S*-RNase loci are marked in dark red on the grey panels representing Chr03 and Chr05. (f) Protein alignment of the four ‘Pru *S*-locus’ *S*-RNase alleles (*pS1=hap1g16438.t1*, *pS2=hap2g17313.t1*) with Pruavi-S1-RNase reference protein. Red triangle denotes K→T mutation in active site of C3 conserved domains. For domain annotations see Supplemental Fig. S5.

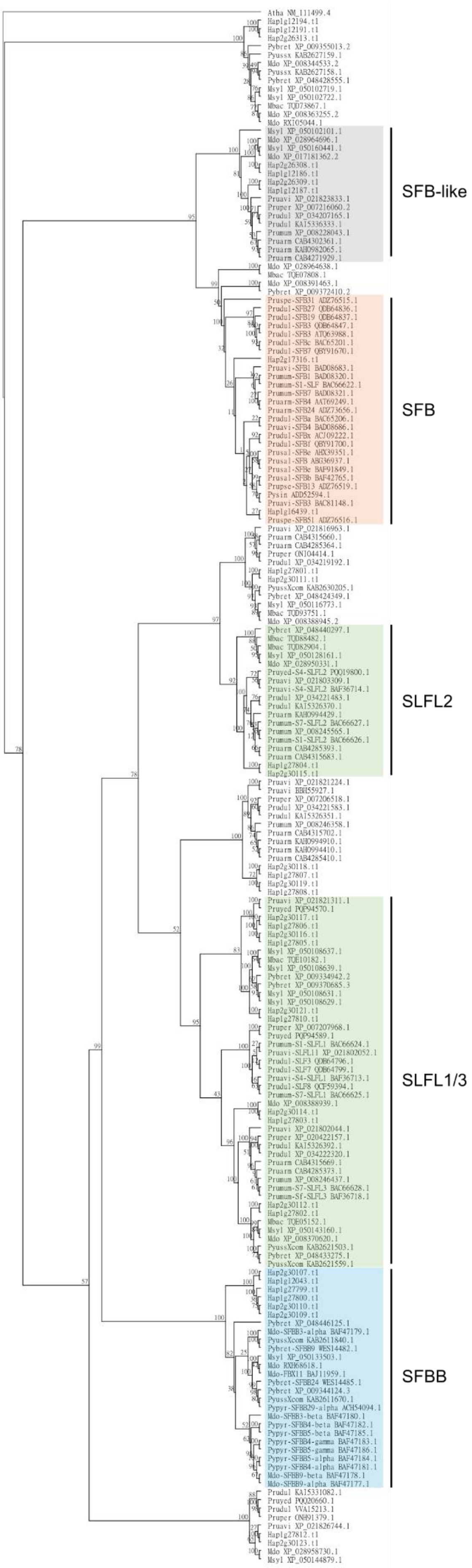

Supplemental Fig. S7. Phylogenetic analysis of *S*-locus F-box proteins.

Phylogenetic analysis of *S*-locus F-box proteins from *Gillenia*, *Prunus*, and Maleae species. Clade nomenclature follows[1] and shows lineages of S-locus F-Box (SFB; orange), SFB-like (grey), S-Locus F-box Like (SLFL; green) and S-locus F-Box Brothers (SFBB; blue). Maximum likelihood bootstrap values from 100 datasets shown at branches. NCBI codes for *Prunus* and Maleae proteins, *Gtr*.v2 gene model IDs for *Gillenia* proteins.

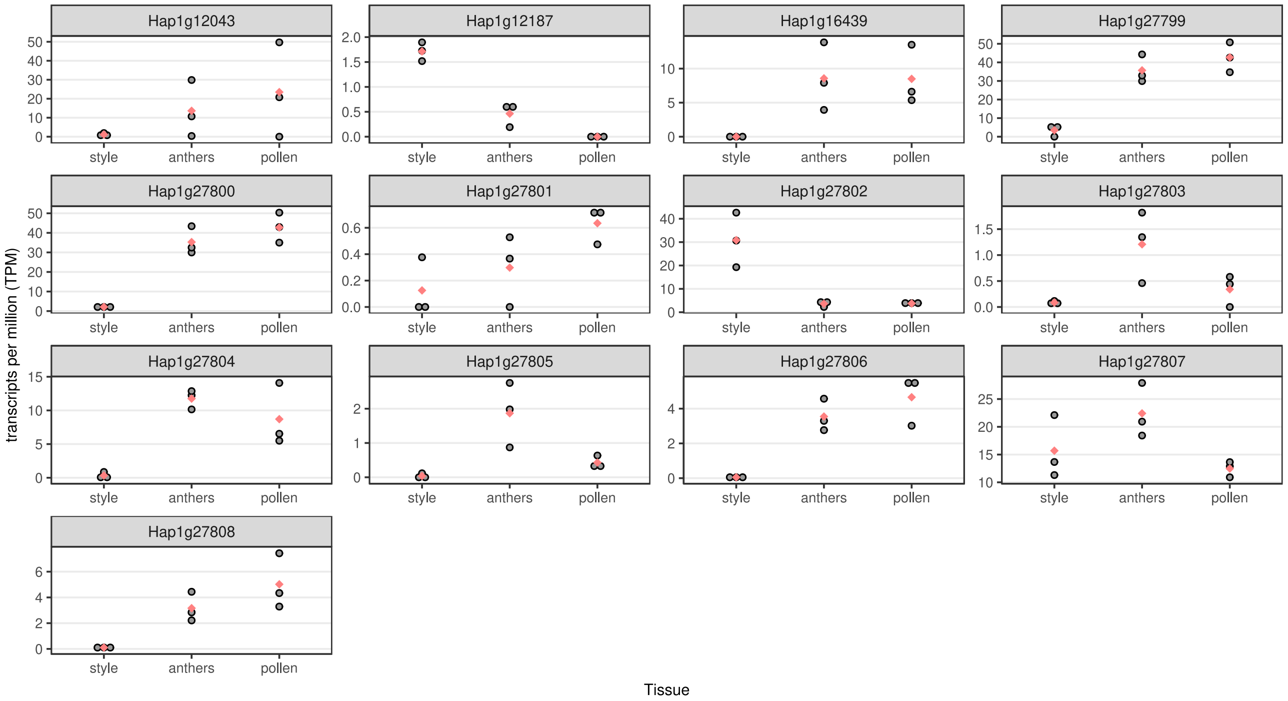

Supplemental Fig. S8. Dot plots showing expression of *Gillenia S*-locus F-box genes.

Gene expression in style/stigma, stamen, and germinated pollen, shown as transcripts per million (TPM), grey circles represent replicates, pink circles represent mean. *SFBB* lineage genes are *Hap1g12043*, *Hap1g27799* and *Hap1g27800*; *SLFL* lineage genes are *Hap1g27801*, *Hap1g27802*, *Hap1g27803*, *Hap1g27804*, *Hap1g27805*, *Hap1g27806*, *Hap1g27807*, and *Hap1g27808*; *SFB* lineage gene is *Hap1g16439*; *SFB*-like lineage gene is *Hap1g12187*.

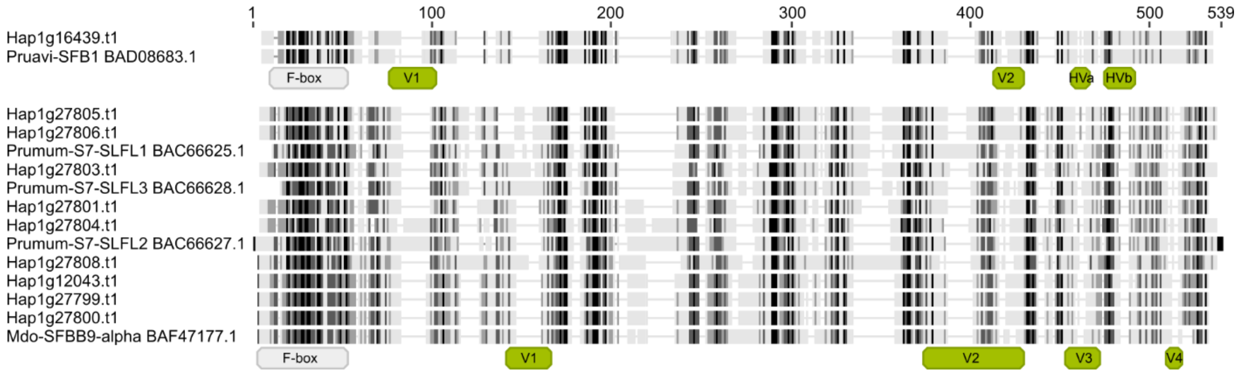

Supplemental Fig. S9. Protein sequence alignment of *Gillenia S*-locus F-box proteins.

Protein sequence alignment of putative *Gillenia S*-locus F-box proteins showing predominant pollen/stamen expression, with representative *Prunus* and Maleae proteins. Domain annotations[5,6] are V1-4, Variable domain 1-4; HVa-b, Hypervariable domain a/b.

a

	pSFB3	pSFB4	pSFB5	pSFB1	pSFB2
pSFB3		99.9%	82.8%	82.8%	83.3%
pSFB4	99.9%		82.8%	84.3%	83.3%
pSFB5	82.8%	82.8%		82.4%	84.9%
pSFB1	82.8%	84.3%	82.4%		97.8%
pSFB2	83.3%	83.3%	84.9%	97.8%	

b

	pSFB5	pSFB3	pSFB4	pSFB1	pSFB2
pSFB5		74.2%	74.2%	76.5%	78.7%
pSFB3	74.2%		100%	78.9%	77.9%
pSFB4	74.2%	100%		78.7%	77.9%
pSFB1	76.5%	78.9%	78.7%		97.1%
pSFB2	78.7%	77.9%	77.9%	97.1%	

c

	mSFBBab1	mSFBBab3	mSFBBab2	mSFBBc3	mSFBBc1	mSFBBc2
mSFBBab1		100%	99.9%	93.7%	93.7%	93.6%
mSFBBab3	100%		99.9%	93.7%	93.7%	93.6%
mSFBBab2	99.9%	99.9%		93.6%	93.6%	93.5%
mSFBBc3	93.7%	93.7%	93.6%		99.9%	99.9%
mSFBBc1	93.7%	93.7%	93.6%	99.9%		99.9%
mSFBBc2	93.6%	93.6%	93.5%	99.9%	99.9%	

d

	mSFBBab1	mSFBBab2	mSFBBab3	mSFBBc1	mSFBBc2	mSFBBc3
mSFBBab1		99.7%	100%	86.9%	86.6%	86.9%
mSFBBab2	99.7%		99.7%	86.6%	86.4%	86.6%
mSFBBab3	100%	99.7%		86.9%	86.6%	86.9%
mSFBBc1	86.9%	86.6%	86.9%		99.7%	100%
mSFBBc2	86.6%	86.4%	86.6%	99.7%		99.7%
mSFBBc3	86.9%	86.6%	86.9%	100%	99.7%	

Supplemental Fig. S10. Sequence diversity within *Gillenia S*-locus F-box alleles.

Nucleotide (a,c) and protein (b,d) distance matrices showing percent identity for the putative *S*-locus F-box alleles of the ‘Pru *S*-locus’ *SFB* gene *hap1g16439* (a,b) and ‘Mal *S*-locus’ *SFBB* genes *Hap1g27799* and *Hap1g27800* (denoted *mSFBBab*) and *Hap1g12043* (denoted *mSFBBc*) (c,d). Mutations sometimes occurred outside transcribed region.

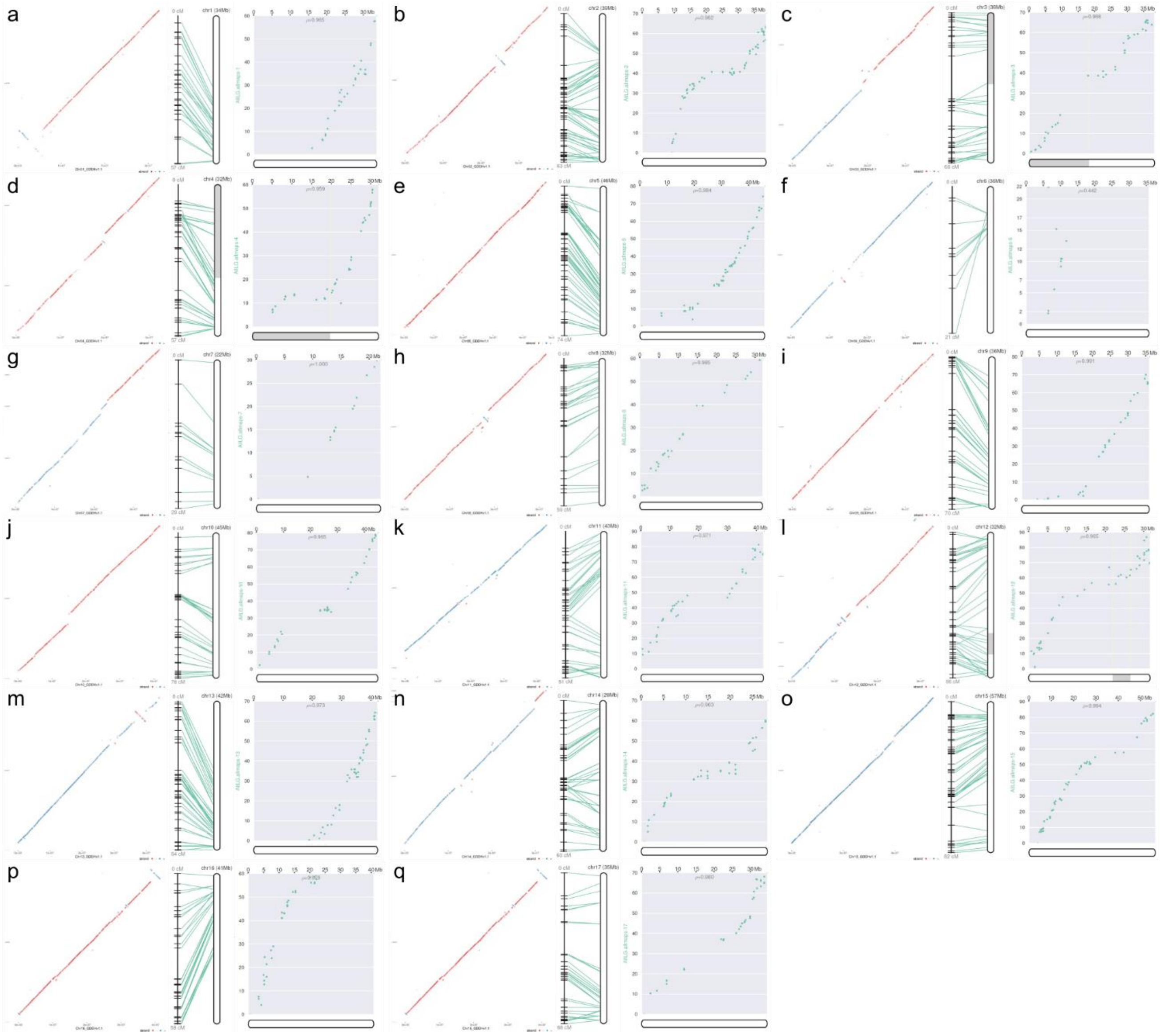

Supplemental Fig. S11. *Malus* **‘**M9’ genome synteny *versus* ‘Golden Delicious’ GDDHv1.1 genome.

(a-q) Haplotype 1 Chr01-Chr017, (r-ah) Haplotype 2 Chr01-Chr017. For each figure, Left, MUMmer alignment dot plots of ‘M9’ hifiasm assembly and the apple reference genome of 'Golden Delicious' (double haploid GDDH13v1.1)[2] using alignments 18 Kb or larger. Red dots represent positive strand alignments while blue dots represent negative strand alignments. Middle and right, CMAP-style plots with lines connecting the physical positions on the reconstructed chromosome and the ‘M9’ map positions.

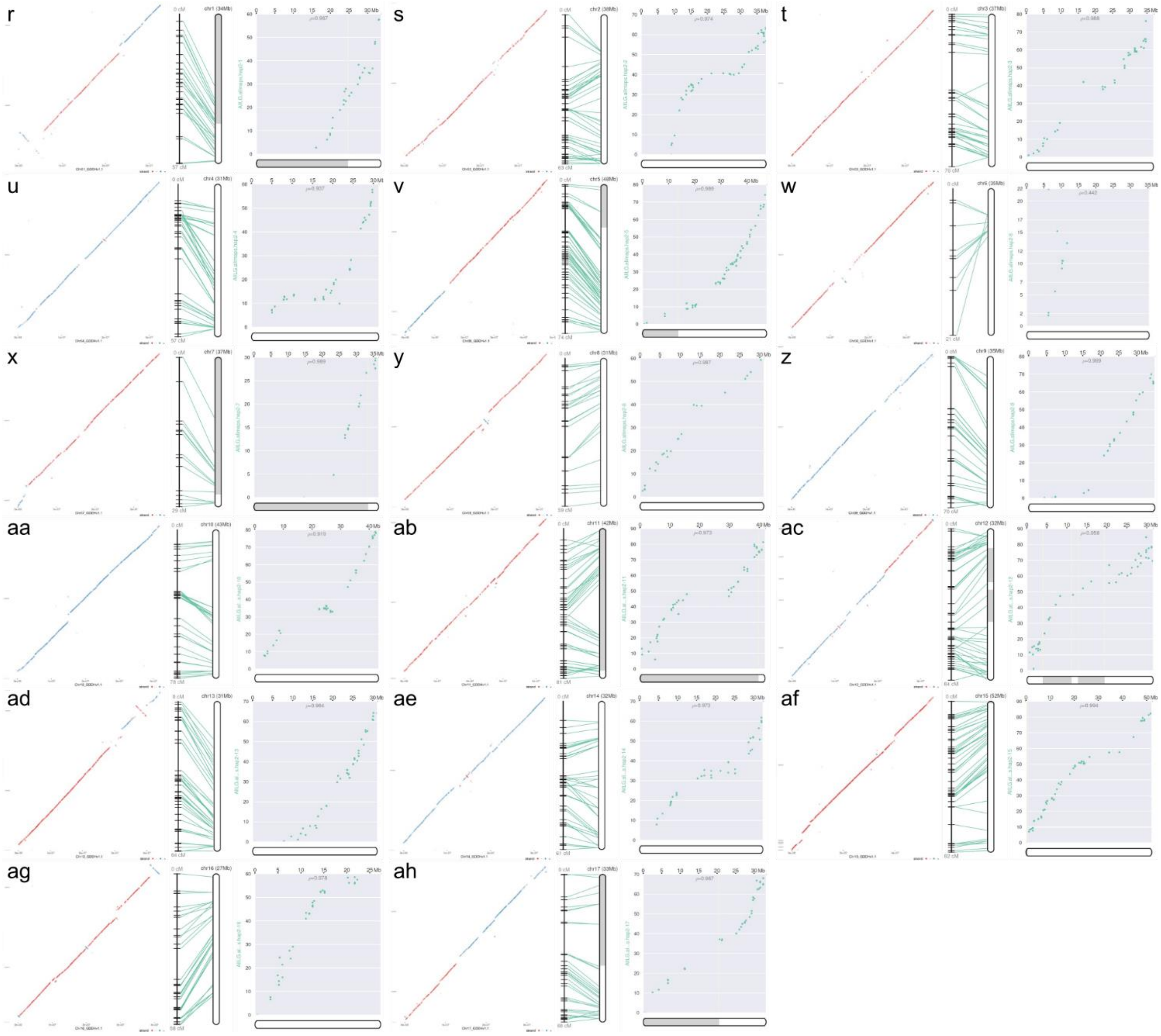

Supplemental Fig. S11 cont. *Malus* **‘**M9’ genome synteny *versus* ‘Golden Delicious’ GDDHv1.1 genome.

(a-q) Haplotype 1 Chr01-Chr017, (r-ah) Haplotype 2 Chr01-Chr017. For each figure, Left, MUMmer alignment dot plots of ‘M9’ hifiasm assembly and the apple reference genome of 'Golden Delicious' (double haploid GDDH13v1.1)[2] using alignments 18 Kb or larger. Red dots represent positive strand alignments while blue dots represent negative strand alignments. Middle and right, CMAP-style plots with lines connecting the physical positions on the reconstructed chromosome and the ‘M9’ map positions.

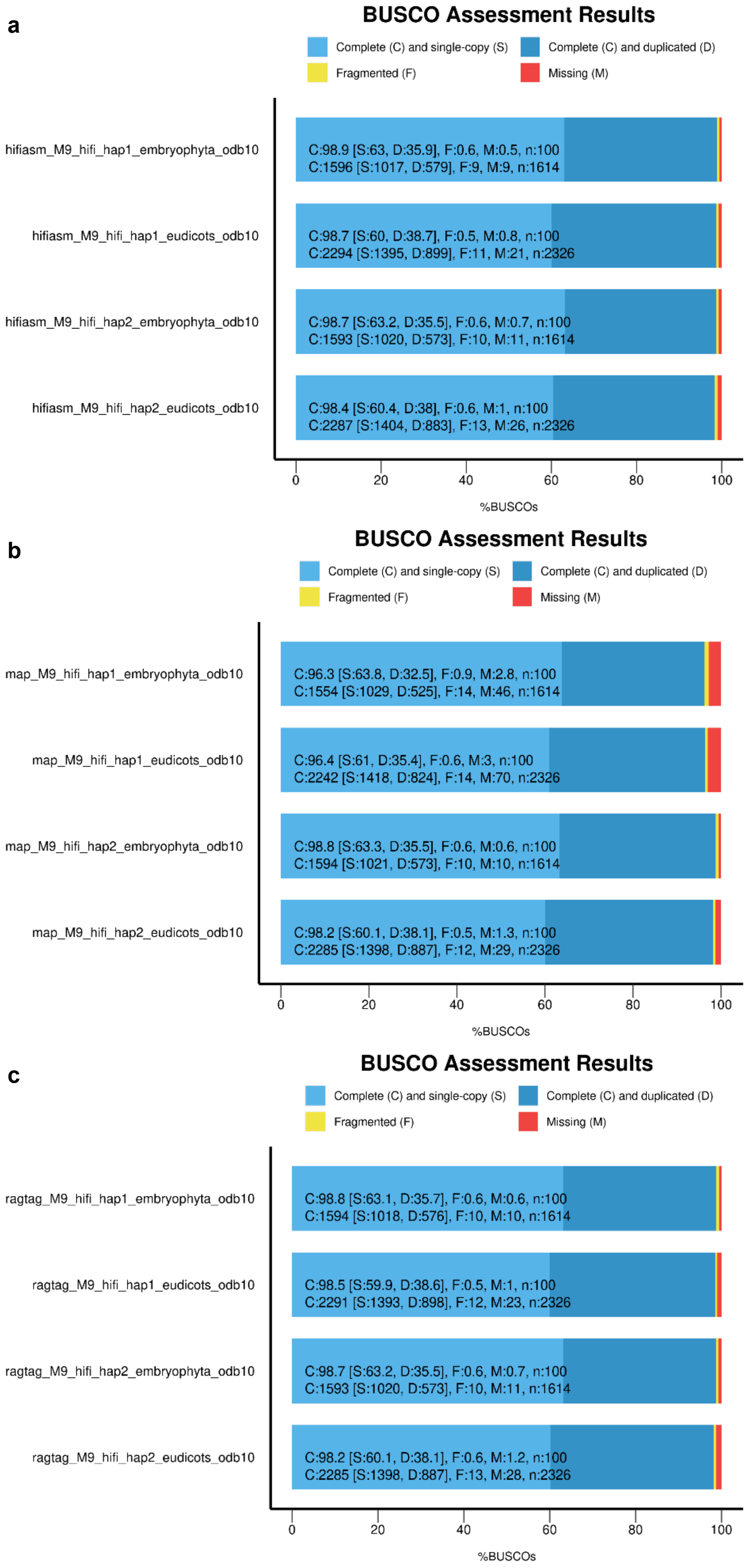

Supplemental Fig. S12. BUSCO scores for *Malus* ‘M9’ genome.

Busco scores of ‘M9’ rootstock genome using the embryophyta_odb10 and the eudicots_odb10 gene datasets. (a) Busco scores of ‘M9’ hifiasm genome assembly. (b) Busco scored of ‘M9’ genome after anchroing to the ‘M9’ genetic map. (c) Busco scores of ‘M9’ genome after scaffolding against the apple reference genome (‘Golden Delicious’ double haploid GDDH13v1.1)[2].

a
b
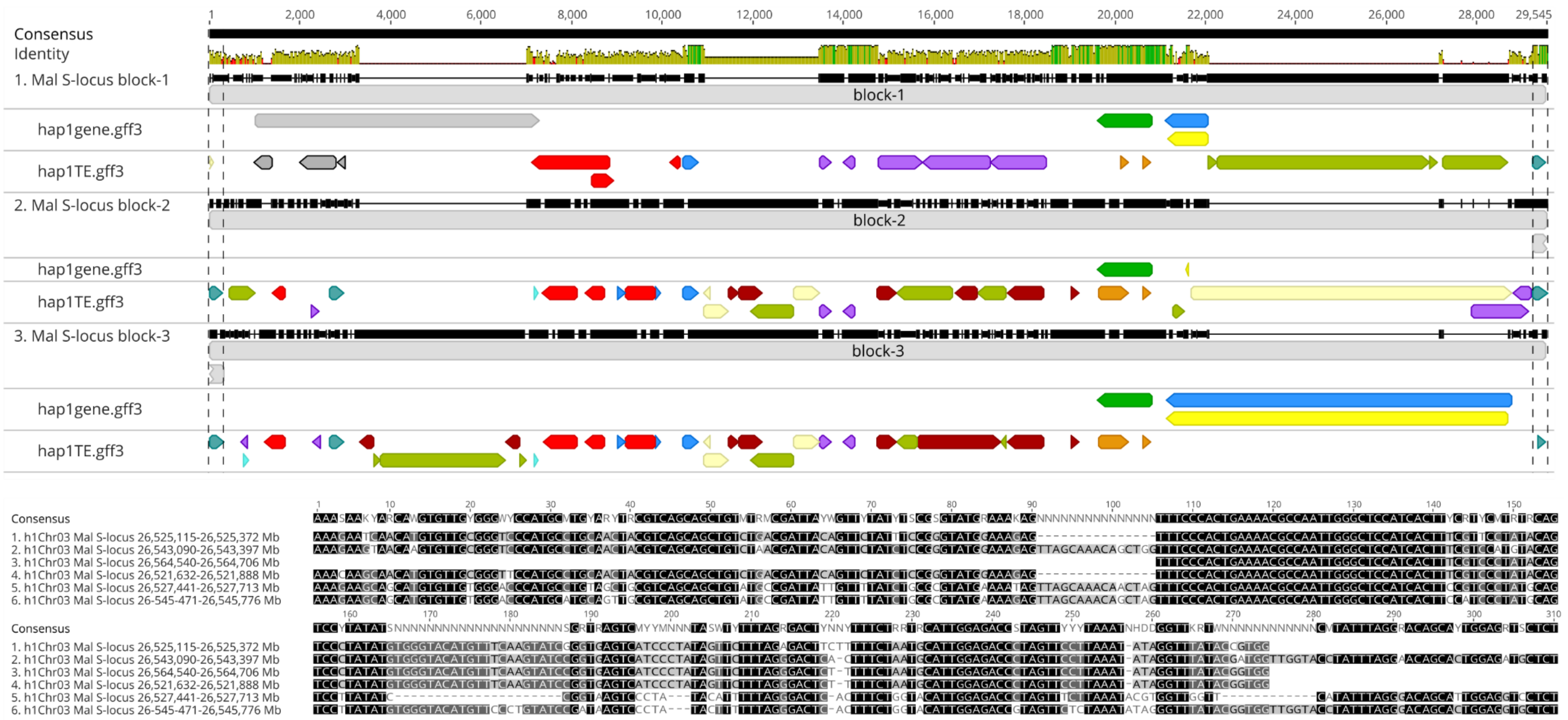

Supplemental Fig. S13. Details of the segmental duplications at *Gillenia* Chr03 'Mal *S*-locus'

(a) MAFFT alignment of the three segmental duplications identified within the *Gillenia* v2 Chr03 'Mal *S*-locus' showing alignment of repeat elements belonging to the same TE family as determined by the EDTA pipeline (teal triangles in hap1TE.gff3 track) at left and right borders of segmental blocks 2 and 3, and right border of segmental block 1. Annotations on the hap1gene.gff3 track are *SFBB* (green), *S-RNase* (block-3) or non-coding *S-RNase* (block-1) (blue), gene with no known S-locus function (grey), RNase_T2 domains (block 1-3) (yellow). Annotations on the hap1TE.gff3 track are LTR annotations are *Ty1/Copia* and *Ty3/Gypsy* (green), LTR retrotransposons (dark red). TIR-TE annotations are hAT (light red), PIF/Harbinger (purple), Mutator (cream), CACTA (light blue). Other-TE annotations are LINE elements (blue) and repeat regions (orange from the same TE family, grey from additional different TE families). (b) Clustal Omega alignment of all six 'repeat region' sequences within the Chr03 'Mal *S*-locus' determined to belong to the same TE family as determined by the EDTA pipeline (teal triangles in (a)).

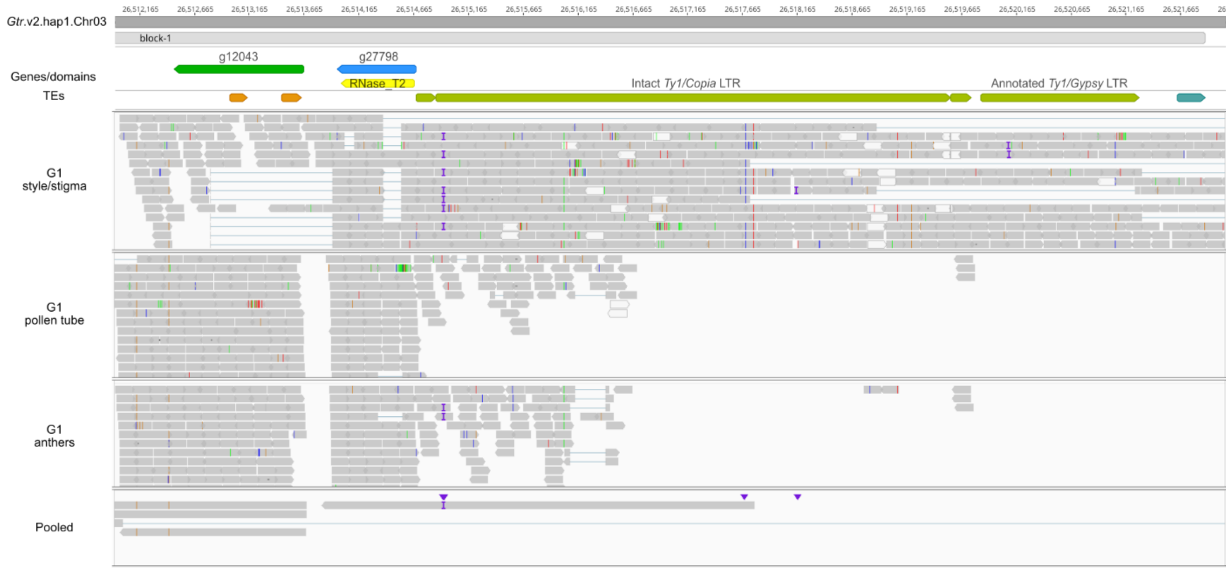

Supplemental Fig. S14. Alignment of short RNA-seq and long IsoSeq reads at the LTR of interest

IGV graphical representation of read alignment to Chr03 'Mal *S*-locus' (26,512,000-26,522,000). Short RNA-seq reads are from G1 individual, for style/stigma, germinated pollen tube, and anther/filament tissues, and long IsoSeq reads are pooled from all individuals and all tissues (style/stigma, pollen tube, anther/filament). Alignment shows reads from all sources aligning along part or all of the intact *Ty1/Copia* LTR element and reads in stigma/style tissues aligning with the annotated *Ty1/Gypsy* LTR element. Annotations follow Fig. 6.

References

1 Aguiar, B. *et al.* Convergent evolution at the gametophytic self-incompatibility system in *Malus* and *Prunus*. *PLoS ONE* **10**, e0126138 (2015).

2 Daccord, N. *et al.* High-quality *de novo* assembly of the apple genome and methylome dynamics of early fruit development. *Nature Genetics* **49**, 1099-1106 (2017).

3 Ireland, H. S. *et al.* The *Gillenia trifoliata* genome reveals dynamics correlated with growth and reproduction in Rosaceae. *Horticulture Research* **8**, 233 (2021).

4 Verde, I. *et al.* The Peach v2.0 release: high-resolution linkage mapping and deep resequencing improve chromosome-scale assembly and contiguity. *BMC Genomics* **18**, 225 (2017).

5 Ikeda, K. *et al.* Primary structural features of the S haplotype-specific F-box protein, SFB, in *Prunus*. *Sexual Plant Reproduction* **16**, 235-243 (2004).

6 Sassa, H. *et al.* *S locus F-box brothers*: multiple and pollen-specific F-box genes with S haplotype-specific polymorphisms in apple and Japanese pear. *Genetics* **175**, 1869-1881 (2007).
